# Current Medical Controversies in Zollinger–Ellison Syndrome

**DOI:** 10.3390/biomedicines13123051

**Published:** 2025-12-11

**Authors:** Robert T. Jensen, Irene Ramos-Alvarez, Jeffrey A. Norton

**Affiliations:** 1 Digestive Diseases Branch, National Institute of Diabetes and Digestive and Kidney Diseases, National Institutes of Health, Bethesda, MD 20892, USA; irene.ramosalvarez@nih.gov; 2 Department of Surgery, Stanford University Hospital, Stanford, CA 94305, USA; janorton@stanford.edu

**Keywords:** chemotherapy, gastric carcinoids, gastrinoma, hypergastrinemia, lifelong acid antisecretory drug treatment, MEN1, pancreatic neuroendocrine tumor/neoplasm syndrome, PRRT, somatostatin receptor imaging, Zollinger–Ellison syndrome

## Abstract

**Purpose**: Zollinger–Ellison syndrome (ZES) is the most frequent, functional, malignant pancreatic neuroendocrine tumor syndrome (pNET), which is due to ectopic secretion of gastrin by a pNET/NET (i.e., gastrinomas) resulting in severe, refractory acid-peptic disease (ulcer, GERD). ZES has several unique management features, which lead to a number of unresolved controversies. **Areas covered**: Whereas both medical and surgical controversies exist, they have not been examined in detail for some time. This review contains an analysis of a number of the main current, medical controversies that are unresolved in ZES patients, including insights into the basis of these controversies and possible insights into their resolution from recent studies in patients with gastrinomas or from recent studies in other pNET syndromes or other neuroendocrine tumors (NETs). These include the following: controversies in the long-term control of acid secretion and acid antisecretory drug side-effects; controversies related to the difficulty in making the diagnosis of ZES; nonsurgical MEN1/ZES controversies related to the management of gastric carcinoids (Type II); nonsurgical MEN1/ZES controversies related to whether genotype–phenotype correlations exist in MEN1 patients including MEN1/ZES patients; nonsurgical MEN1/ZES controversies related to the roles of imaging/tumor localization in MEN1 patients for gastrinomas/pNETs in their initial/follow-up management; controversies related to the role of non-surgical tumor ablation for treatment of ZES/gastrinomas; and controversies related to medical treatment selection for advanced, metastatic disease in patients with ZES/gastrinomas/other malignant pNETs. **Conclusions**: In this paper, the basis for the development of each of these unique ZES-related controversies is discussed and insights into progress that could lead to their resolution are reviewed.

## 1. Introduction

Zollinger–Ellison syndrome (ZES), first described in 1955 [1], is characterized by gastric acid hypersecretion due to ectopic secretion of gastrin by a neuroendocrine tumor (NET) (gastrinoma) resulting in severe peptic ulcer disease (PUD)/gastroesophageal reflux disease (GERD), which is often refractory to standard PUD treatments [2,3,4,5,6,7,8,9,10,11]. All patients with ZES, similarly to patients with other functional pancreatic neuroendocrine tumor (F-pNET) syndromes, have two major treatment problems—control of the functional syndrome due to the ectopically secreted hormone (i.e., gastrin) and treatment directed at the pNET itself, because in most F-pNET syndromes, except for insulinomas, the pNETs are malignant in >50% of cases [12,13,14,15,16,17]. While successful surgical resection of the pNET would solve both treatment requirements, gastrinomas, unfortunately, similar to other F-pNET syndromes, but different from insulinoma, are not curable in >50% of the cases, primarily because of the presence of advanced disease or multifocal (Multiple Endocrine Neoplasia-type-1[MEN-1]) disease [5,18].

The inability to surgically cure most patients with gastrinomas has led to a number of controversies involving both medical and surgical issues, some of which are shared in the long-term treatment of other F-pNET, but also a number which is unique to patients with ZES. In the past, a number of reviews have attempted to highlight aspects of these controversies [19,20,21,22,23], but there have been a number of recent advances/studies [6,16,24,25,26,27,28,29,30,31,32,33,34,35,36,37,38,39,40,41,42,43,44,45,46] involving both patients with gastrinomas or with other pNET or GI-NETs (carcinoids), which have provided important insights that pertain to these controversies in ZES/gastrinoma patients. In this paper, we review a number of the most important current controversies in the medical management of patients with ZES incorporating the results of these recent studies. In a later study a similar analysis is planned for the specific analysis of the unique controversies related to surgical aspects of gastrinomas and the effects of recent studies on gastrinomas and other NETs on providing insights into possible resolution of these surgical controversies.

## 2. Current Medical Controversies

### 2.1. Controversies in Long-Term Control of the Acid Hypersecretion of ZES Patients and the Increasingly Reported Acid Antisecretory Drug Side-Effects

#### 2.1.1. Background to Current ZES Acid-Secretory Controversy

Patients with ZES have prominent gastric acid hypersecretion secondary to the hypergastrinemia with basal acid outputs (BAO) when untreated, averaging 4-fold the normal rate, reaching up to >10-fold increase in some patients [47,48], because of the trophic effects of gastrin on gastric parietal cells resulting in a 4–6-fold increase in parietal cell mass [5], which in turn results in an elevated maximal acid output (MAO) levels [47,48], as well as the BAO. From the initial descriptions of ZES patients it became quickly apparent that if the acid hypersecretion was not rapidly controlled these patients had very high morbidity and mortality due to uncontrolled acid-peptic complications [1,2,3,49]. Furthermore, even today, because the majority of ZES patients are not surgically curable, similarly to a number of other non-insulinoma F-pNET syndromes, primarily due to the presence at diagnosis of advanced or multifocal disease, most patients require lifelong acid antisecretory medications to control their gastric acid hypersecretion [27,28,33,40]. The percentage requiring life-long medical treatment is further accentuated in patients with ZES, because it has the highest percentage of patients of any F-pNET syndrome due to the presence of the inherited disorder, Multiple Endocrine Neoplasia type 1 (MEN1) (MEN1/ZES), which is 20–25% of all ZES patients [50,51,52,53]. MEN1/ZES patients have, in the vast majority, multiple, frequently small gastrinomas in the duodenum associated with >50% having lymph node metastases, resulting in them having a very low surgical cure rate (<0–<5%) with the standard surgical operations that are currently recommended; however, they do not include aggressive surgical resections such as a Whipple procedure [18,28,54,55,56,57,58], which is not routinely recommended because of its increased morbidity [28,58]. Currently, proton pump inhibitors (PPIs) have become the antisecretory drugs of choice to treat the acid hypersecretion in these patients, largely replacing the use of histamine H_2_-receptor antagonists (H_2_R), because of their increased potency and long duration of actions [6,33,59,60,61,62,63,64,65,66,67,68,69,70].

Prior to the development of PPIs, H_2_Rs were the only effective medical therapy available at this time; however, there was an increasing number of reports of failure to control the acid secretion in many ZES patients with H_2_Rs [59,71,72,73,74,75,76,77]. This failure was attributed to the fact that H_2_Rs had a relatively short duration of action (4–8 h) and relatively low potency in many ZES patients; thus, many ZES patients required frequent, high doses to adequately control their acid secretion [47,59,71,78]. In most patients the H_2_R dosages had to be individually titrated by measuring acid output prior to the next drug dose and using established criteria of acid control, which was frequently not performed [59,79,80].

#### 2.1.2. Current Acid-Secretory ZES Controversy

Whereas both the initial controlled studies with all the different PPIs (omeprazole, lansoprazole, esomeprazole, pantoprazole, rabeprazole), as well as a number of subsequent studies of series of patients with both sporadic ZES and ZES/MEN1 patients, reported that PPIs could control the acid hypersecretion in nearly all patients, except those who would or could not regularly take the medication, both initially and long-term [33,59,65,67,81,82,83,84,85,86,87,88,89,90,91,92,93,94,95]. However, recently there has been an increasing number of primarily case reports regarding difficulty in successfully controlling acid hypersecretion, mainly long-term in ZES and MEN1/ZES patients [38,41,96,97,98,99,100,101,102,103,104,105,106,107,108,109,110,111,112,113,114,115,116,117,118,119,120]. In most of these case reports the difficulty with PPI treatment was a failure to adequately control symptoms or complications of ZES with the doses of PPIs used, even when the doses were increased in some cases. In some cases [101,108,113,119] the difficulty was the development of adverse side-effects of the PPI (i.e., hypomagnesemia) which led to either discontinuation of PPI treatment or a more complicated acid antisecretory treatment program occurred, which will be discussed in a following paragraph in more detail.

**Figure 1:**
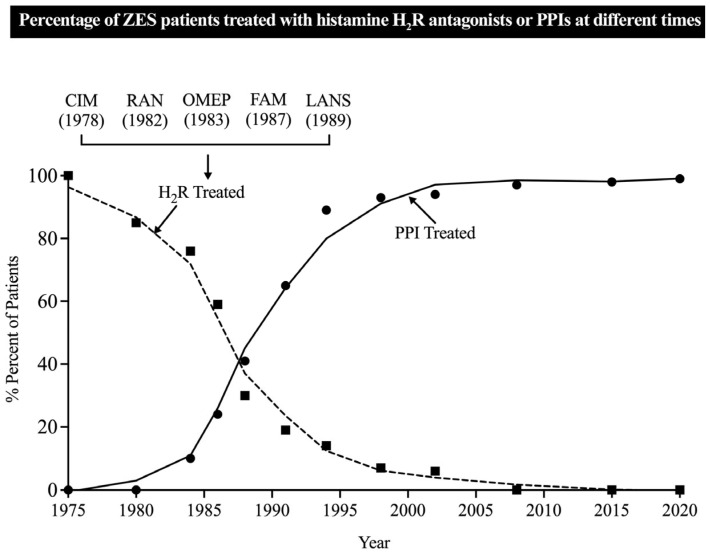
Antisecretory drugs used at different time in 303 patients with ZES treated at NIH. Listed are the percentage of patients treated with either histamine H_2_-receptor antagonists (cimetidine, ranitidine, famotidine, nizatidine) or PPIs (omeprazole, lansoprazole, pantoprazole) over time periods. Data are reported as percentage of patients during the given time period treated with either group of drugs. Figure/data are from [33,121] and are freely available as authors from US government agency NIH.

The above recent case reports of failed PPI treatment differ markedly from the results reported in a recent NIH study [33] of long-term control of acid hypersecretion in 303 ZES patients treated for a mean of 13.7 yrs (range 0.08–48.1 yrs), in which medical control of the hypersecretion was successful in all patients both acutely and long-term. In the above study [33], the original use of H_2_Rs in these patients was rapidly replaced by PPI treatment in the 1980–2000 time period (Figure 1).

**Figure 2:**
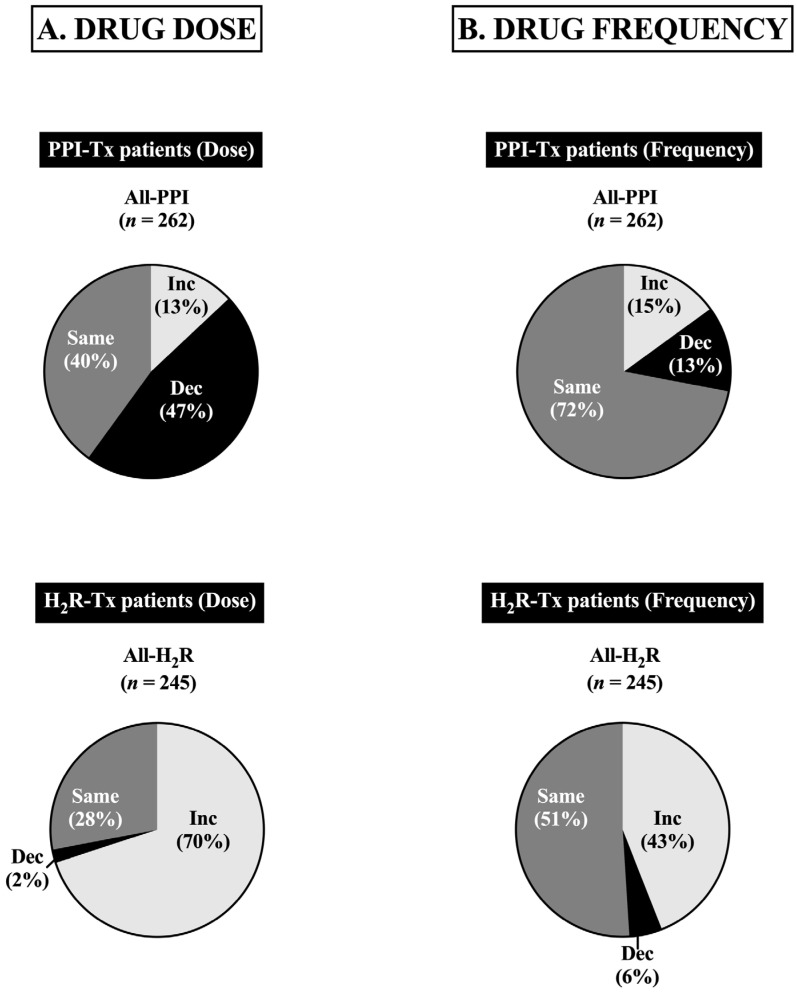
(**A**) Pie charts showing distribution of changes in drug dosage from first to last dose in 303 ZES patients treated for a mean of 10.2 years (range up to 32 yrs) with either a histamine H_2_-receptor antagonist or proton pump inhibitor. These data show the percentage of ZES patients treated with either a histamine H_2_-receptor antagonist or proton pump inhibitor at any time. For the PPI users the mean times between the first and last dose for the total were 9.78 ± 0.35 yrs. For the H_2_-R group the 1st to last dose time was 6.38 ± 0.32 yrs. Figure/data modified from [33]. (**B**) Pie charts showing distribution of changes in daily drug frequency with treatment with either a histamine H_2_-receptor antagonist or proton pump inhibitor from first to last dose in 303 ZES patients treated for a mean of 10.2 years (range up to 32 yrs). Data show the percentage of ZES patients treated with either histamine H_2_-receptor antagonist or proton pump inhibitor at any time. The duration between the first and last daily dose used for comparing daily dose frequency is the same as listed for the daily dose comparison in Figure 2A legend. Figure/data are modified from [33] and are freely available as authors from US government agency NIH.

In the study [33], it is shown that with time the PPI daily dose (Figure 2A), as well as its daily PPI dose frequency (Figure 2B), could actually be decreased in 47% and 13% of the patients, respectively, which was in marked contrast to what was seen with changes in H_2_R dosage in time (Figure 2A), in which the daily dose was able to be decreased in only 2% of the patients and 6% in frequency (Figure 2B); in fact, the daily H_2_R dose had to be increased in 70%, and in 43% the daily H_2_R dose frequency had to be increased (Figure 2A,B).

**Figure 3:**
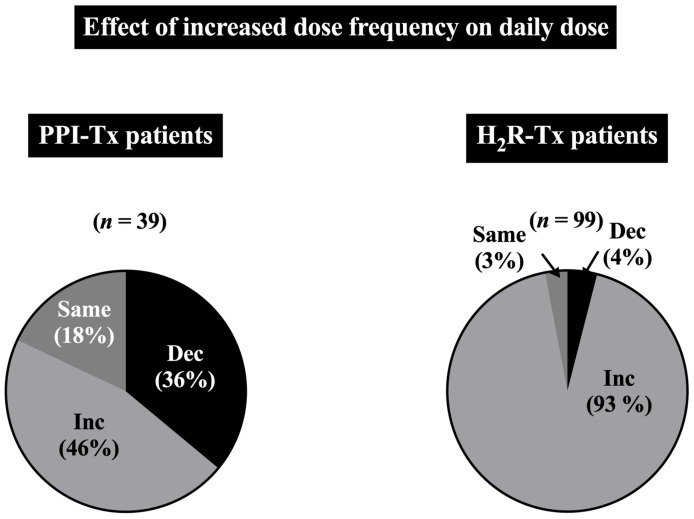
Percentage changes in total drug daily dose in ZES patients who had an increased frequency of dosing during treatment with either an H_2_R antagonist or PPI are shown. The duration between the first and last daily dose used for comparing daily dose frequency is the same as the one listed for the daily dose comparison in Figure 2A,B legend above. Figure/data are modified from [33] and are freely available as authors from US government agency NIH.

In this long-term study, which was performed by titrating each drug dose individually for each patient each year, in the 303 ZES patients it was found that the daily drug dosage frequency could have a marked effect on total daily drug dose required, which differed for PPIs and H_2_Rs (Figure 3).

As shown in Figure 3, comparing the first and final total drug dose in patients who had their daily antisecretory dose frequency increased with PPI-treated patients resulted in 36% requiring a lower daily dose and an overall increase in daily dose in 46%, whereas 93% of H_2_R-treated patients required an increased daily dose and only 4% showed a decrease in daily dose (Figure 3). These drug dosage changes could only be determined by directly assessing for each patient the effect on the acid secretory rate after each drug dose/frequency change. The difference in results from this recent large study [33], as well as the previous shorter-term controlled studies of PPIs in ZES reporting high success in controlling acid secretion in ZES patients, from the above case reports showing a high rate of drug failure to control the acid hypersecretion, is whether systematic acid secretory testing using established criteria of drug efficacy were routinely used to individually titrate the PPI antisecretory dose during both the initial treatment and on follow-up visits [33]. Unfortunately, gastric acid secretory studies are no longer routinely performed in most hospitals; therefore, the above results suggest that resolution of this controversy is best resolved by recommending that ZES patients for best care should be referred to a specialty center that has either the capability of assessing acid control status of antisecretory drugs or has developed other local criteria that allow long-term successful monitoring of the acid secretory control. These include centers having expertise in performing a careful history of acid-peptic/GERD symptoms, diarrhea, and overall health; the performance of regular UGI endoscopic assessments to establish lack of PUD/GERD findings on anti-secretory drugs; and a possible assessment of drug-control acid secretory rates perhaps at the time of endoscopic evaluation, as recently described [122]. This recommendation is necessary at this point because, unfortunately, at present, although a number of large centers report satisfactory control of the acid hypersecretion in ZES patients with acid antisecretory drugs, which in many cases do not involve systematic acid secretory studies, except for the NIH study above, there are no prospective studies that clearly define the methodology used and patient follow-up that could be used by other groups that might not have acid secretory studies available.

The question of the long-term safety/side-effects on lifetime treatment with PPIs may become an increasingly controversial issue in ZES patients, as it already is for chronic treatment of patients with more common disorders requiring lifelong acid suppressive treatments, such as patients with chronic GERD [114,123,124,125,126,127,128,129,130,131,132,133,134,135]. These safety issues have been heightened by numerous papers/reports, primarily from epidemiological or observation studies of potential serious side-effects linked to PPIs usage in patients primarily with chronic GERD often requiring life-long acid antisecretory treatment and including an increased occurrence of bone fractures and bone problems such as effects on dental implants; chronic renal disease; nutritional/drug malabsorption (vitamin B_12_, iron, calcium, magnesium); cardiovascular disease (CVS) (CVS mortality, myocardial infarction, stroke); CNS abnormalities (dementia); increased infections (Clostridia, bacterial infections with liver disease); lung diseases (pneumonia); interference with metabolism of important therapeutic agents/drugs; and effects on neoplastic processes including the development of carcinoid tumors and other neoplasms [114,123,124,125,126,128,129,130,131,132,133,134,135,136]. The exact mechanisms that may be involved in PPIs causing most of these side-effects are not clear; however, in the case of nutrient malabsorption, which is discussed below for V12 deficiency in detail, it is due to the PPI-induced hypo-/achlorhydria and is similar for PPI-induced iron deficiency [127,133,137,138]. For PPI-induced renal disease a number of mechanisms have been proposed and are discussed in a few recent papers [114,123,124,125,126] by these studies, and some randomized control studies do not show increased incidence of side-effects [123,139]; nevertheless, the increased concern persists. A number of these proposed PPI side-effects have been reported in patients with ZES with long-term PPI treatment [38,41,59,101,108,113,119,136] (particularly hypomagnesemia and low vitamin B_12_ stores/vitamin B_12_ deficiency), which have led to difficulties in long term acid control and even the need for total gastrectomy [108]. 

In non-ZES patients (i.e., primarily patients with idiopathic chronic moderate/severe GERD requiring long-term PPI maintenance treatment), numerous case reports, small series, and three meta-analyses report the association of chronic PPI use with the subsequent development of hypomagnesemia, which can be severe and life-threatening [108,114,124,125,126,140,141,142,143,144,145,146]. The exact frequency of occurrence of hypomagnesemia with PPI chronic use is not clear, but its frequency is increased with a pooled risk ratio (RR) in different studies of 1.8 [143], 1.44 [125], and a third study reported a 43% increase in the incidence of hypomagnesemia in patients taking PPIs over matched controls [147] with its occurrence comprised 1% of the adverse PPI events reported to the FDA [148]. When PPI use is stopped, the hypomagnesemia disappears and, in some cases, recurs when the PPIs were restarted [113,136,144,149,150]. Evidence suggests the development of the hypomagnesemia is a general PPI class-effect, because its redevelopment has been reported with the subsequent use of other PPIs, but not when histamine H_2-_receptor antagonists were subsequently used [114,136,144].

The mechanism of the hypomagnesemia is not clear [136,145,151,152,153], with studies generally showing no increased renal secretion, and others suggesting/providing evidence for increased GI losses of magnesium with chronic PPI use [145,151,152,153,154]. In addition, a recent animal study provided evidence and concluded that PPI-induced changes in the gut microbiome are important in the pathogenesis of PPI-induced hypomagnesemia [151]. In prospective PPI studies in NIH ZES patients, only a single case (not reported) of hypomagnesemia was encountered, which resulted in a rate of 0.4% in the >300 patients chronically treated [155]. At present, the rate of occurrence of hypomagnesemia in ZES patients appears low; however, this conclusion is based on prolonged treatment of relatively small numbers of ZES patients and whether with the high doses of PPIs frequently required by ZES patients combined with the prolonged lifelong treatment requirement will result in a higher rate, as suggested by the recent case reports, is also unclear. It is also unclear at present how often the occurrence of hypomagnesemia in ZES patients will be a major management problem, as reported in one ZES patient who developed such refractory hypomagnesemia that a total gastrectomy was required [108]. It is also unclear at present whether substitution of a member of the new class of potent H^+^/K^+^ ATPase inhibitors, the K^+^ channel competitive blockers (P-CAB), such as vonoprazan [156,157,158], could be of help in such patients.

**Figure 4:**
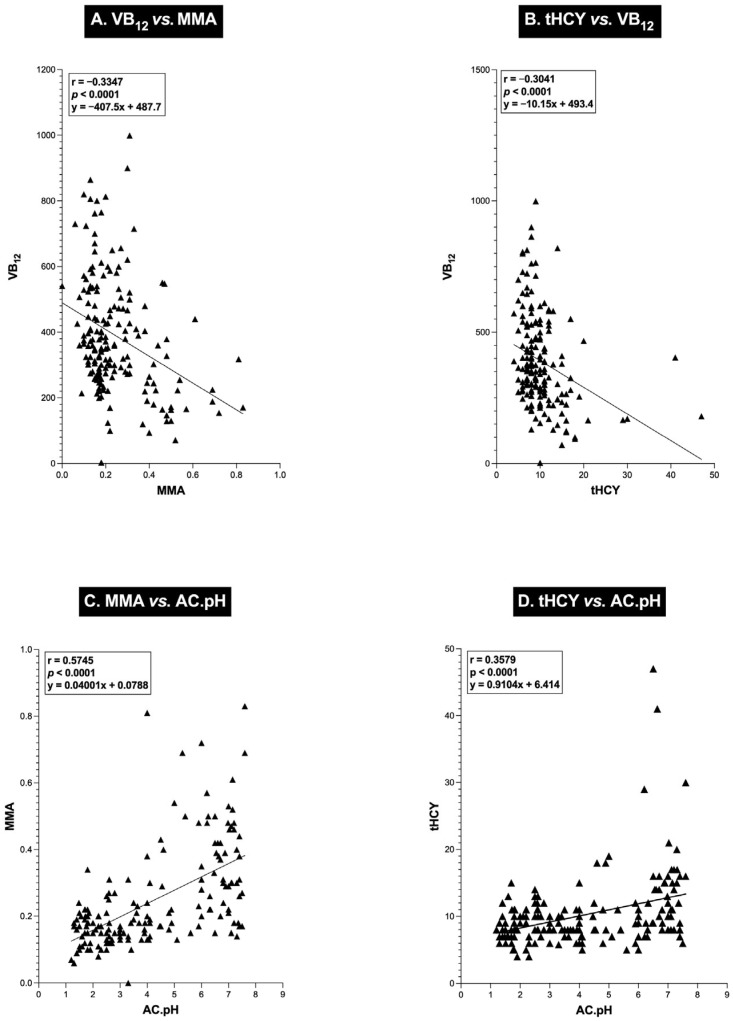
Correlations between NIH patient values for serum MMA or plasma tHCY with serum vitamin B_12_ levels and acid control levels. Each triangle is a value from one patient determined from the same NIH admission. Panel (**A**) (Top). The results show high significant correlations with the increasing serum MMA or plasma tHCY correlating directly with increased pH (decreasing acidity) of the gastric acid control value (Panels (**A**,**C**)) and inversely with the acid output value in mEq/h. (Panels (**B**,**D**)). Figure/data are from [185] and are freely available as authors from US government agency NIH.

Until recently, a major area of controversy and uncertainty existed, with regard to whether the potential safety issue of whether chronic long-term treatment with PPIs in patients with ZES or any group of patients requiring potential long-term/life-time treatment can affect the absorption of the essential nutrient, vitamin B_12_ (VB_12_), which could result in decreases in serum vitamin B_12_ levels/body stores to an extent that vitamin B_12_ deficiency can develop, and if it does this, by what mechanisms PPIs cause this [159,160,161,162,163]. This controversy existed not because this question had not been well-studied, but, instead, because of the differing results in previous studies that have been performed. Most of these previous studies were not performed in ZES patients but were primarily performed in patients taking PPIs long term for moderate to severe idiopathic GERD. A number of these studies reported that there is a long-term treatment effect of PPIs which can result in a decrease in serum VB_12_ levels/body stores, which can result in the development of VB_12_ deficiency [162,164,165,166,167,168,169,170,171,172,173,174,175,176,177,178]. In contrast, a number of other studies did not find this and instead reported no effect of PPIs on VB_12_ stores, and/or no development of VB_12_ deficiency due to the PPI usage was seen [163,179,180,181,182,183]. Two of these studies were in ZES patients [163,184], but differing results/conclusions were reported. Meanwhile, both studies reported with long-term PPI treatment that there was a decrease in serum VB_12_ levels. In one study [184], indirect evidence was provided suggesting this was due to PPI-induced hypo-/achlorhydria; however, in this study, direct evidence was provided by performing studies on VB_12_ body stores to determine whether VB_12_ deficiency actually developed or the relationship of any VB_12_ changed the continued PPI treatment or provided any evidence into the possible mechanisms that might be involved. In the second study [163] it was concluded that the decrease with chronic PPI treatments in the acid hypersecretion of the ZES patients could not account for the change in serum VB_12_ or the VB_12_ deficiency seen in some patients. Recently, the results of long-term study [185] (mean antacid-secretory treatment, 10.2 years [185], [5.6 with PPIs]) of 175 ZES patients from the NIH was reported, in which the serial drug-induced acid-secretory rates were correlated with the presence or absence of development of VB_12_ deficiency determined by serially assessing serum VB_12_ levels proposed to indicate VB_12_ deficiency [186,187,188,189,190,191,192,193,194] and the well-established markers of VB_12_ body stores (blood levels of methylmalonic acid (MMA) and total homocysteine [tHYC]) [187,188,189,190,191,192,193,194,195,196,197,198], as well as correlation with other features of ZES. This study [185] found that over this treatment period of mean 10.2 yrs (5.6 yrs with PPIs) 21% of the ZES patients developed VB_12_ deficiency and that the presence of VB_12_ deficiency did not correlate with any clinical, laboratory, of tumoral feature of ZES, but was associated with a 12-fold lower drug-induced acid-control rate of gastric secretion, a 2-fold higher drug-induced acid control pH, and a higher percentage of patients with drug-induced acid control rates below the required level for activation of pepsin, a key gastric protease essential to liberate food-bound VB_12_ to allow absorption [163,199]. Highly significant inverse correlations (*p* < 0.001) were found between the serum levels of VB_12_ and the serum MMA and plasma tHCY levels (Figure 4A).

In the serum, MMA or tHCY and the acid pH/acid output as well (Figure 4B) were analyzed, as well as the acid pH/output and serum VB_12_ levels while taking long-term acid antisecretory drug treatment [185].

**Figure 5:**
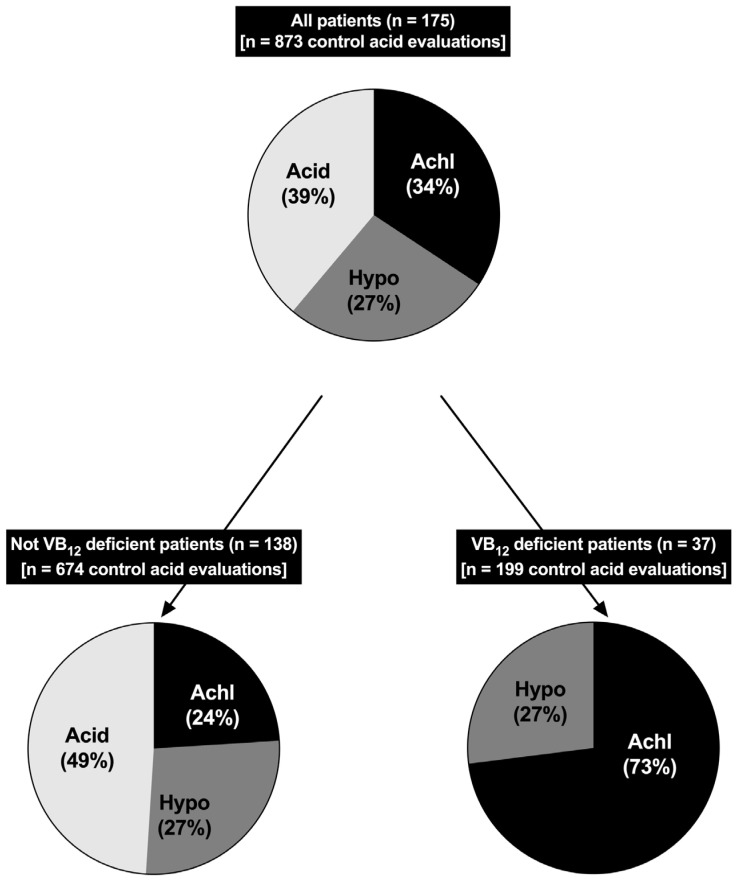
Diagram showing mean gastric acid control value acid output category from all ZES patient admissions [*n* = 873] over a 5-year period (1997–2001) stratified by presence or absence of VB_12_ deficiency. All patients were assigned to one of three categories: the presence of sustained achlorhydria (>50% admission acid control = 0) [Achl], sustained hypochlorhydria (acid control levels from 0.1 to <1 mEq/h->50%) [Hypo], and patients with normal gastric acid present in >50% acid controls ≥1 mEq/h [Acid]. Percentages are the percent of patients in each VB_12_ group [i.e., all pts (*n* = 175); VB_12_ deficient patients (*n* = 37), not VB_12_ deficient patients (*n* = 138)], which were in each of the mean acid control categories. For all patients (*n* = 175), VB_12_-deficient (*n* = 37) and not-VB_12_-deficient groups (*n* = 138) were in the achlorhydric category, respectively (60, 27, 33 patients); in the sustained hypochlorhydria category there were 47, 10, 37 patients; and in the acid category there were 68, 0, 68 patients. The presence of achlorhydria was significantly higher in the vitamin B_12_-eficient category of patients (73% vs. 24%) 604 (*p* < 0.001), but not in the hypochlorhydria category (*p* = 0.99), and the presence of normal acid secretion was significantly higher in the VB_12_-non-deficient than VB_12_-deficient patients (49% vs. 0%) 606 (*p* < 0.001). Figure/data are from [185] and are freely available from authors from US government agency NIH.

In addition, no patient taking H_2_Rs in this study developed VB_12_ deficiency and 92% of all the patients with VB_12_ deficiency had been treated only with a PPI. Furthermore, this study [185] found that over a 5-year period of treatment, the VB_12_-deficient ZES patients had a higher rate of achlorhydria (73% vs. 24%) and a lower rate of normal acid secretion (0% vs. 49%) (Figure 5).

The authors [185] concluded from this study that in ZES patients, the chronic long-term treatment with PPIs results in decreased serum VB_12_ levels and decreased VB_12_ body stores, which can result in VB_12_ deficiency in these patients, which is due to the gastric acid suppressive action of the PPIs. While the PPI-induced decrease in VB_12_ could have a number of important implications for long-term health in these patients, this recent study [185] provides some important insights into how this can be managed. In the conclusion of this study fifteen patients who were not VB_12_ deficient but had low serum levels of VB_12_ took multivitamins containing VB_12_ on their own without altering their PPI dosage. In all the patients the serum VB_12_ level increased by a mean of 34% (*p* = 0.003), with a decrease in 87% of the serum MMA level and a decrease in plasma tHCY levels (indicating an increase in VB_12_ body stores). This occurred because the crystalline VB_12_ in these vitamin tablets is not food-associated and does not require acid activation of proteases for cleavage from food, as does VB_12_ in natural food, and thus can be absorbed in an acid free milieu. This result in the small number of patients suggests that VB_12_ deficiency can be easily avoided in these patients with the concomitant use of oral multivitamins, an observation that will need to be more widely studied.

### 2.2. Controversies Related to the Difficulty in Making the Diagnosis of ZES

#### 2.2.1. Background to Current Controversy in ZES Diagnosis

At present, the best approach that should be routinely used for the diagnosis of ZES is unclear and this is one of the most controversial issues in the management of ZES at present [16,200,201,202]. This controversy has largely arisen for two reasons. The first reason is the demise of routine gastric analysis, which was widely available in the past and was a routine procedure available in most hospitals, to the current state where it is only available in a selected few centers in the US (<5) and in other countries [200,203]. The second reason is related to the widespread use of PPIs in patients with idiopathic GERD/peptic ulcer disease (PUD) in the general population, where it has become the drug class of choice for this disease. These patients generally require chronic and in some cases lifelong marked suppression of gastric acid secretion to adequately control their GERD symptoms, which in many patients can only be accomplished by using PPIs, primarily because of their potency and long durations of action (up to one week) [91,204,205]. The result of marked suppression of the gastric acid secretion in these GERD patients, as well as other non-ZES subjects treated with PPIs, is that 80–100% develop PPI-induced hypergastrinemia [206,207,208,209,210,211,212,213]. Hypergastrinemia develops rapidly after starting PPI treatment (within 5 days); it is a frequent finding among both patients with advanced GERD symptoms as well as normal patients with occasional GERD/indigestion symptoms because PPIs are not only one of the most widely prescribed and overprescribed medications, but they are also now available as over-the-counter medications [214,215,216]. The extent of hypergastrinemia due to PPIs can vary markedly between different patients, with >20% of those taking PPIs in some studies having their FSGs increased >4-fold; FSG levels > 5-fold are not infrequent, with FSG levels even exceeding >10-fold increased—all of which have been reported [206,207,208,209,210,211,212,213]. PPIs treatment is a major problem in the diagnosis of ZES because not only does its use induce hypergastrinemia in non-ZES patients, therefore mimicking ZES, but because of their potency/long-duration of action, in contrast to H_2_R antagonists (nizatidine, famotidine cimetidine, ranitidine); PPIs control symptoms in most ZES patients at conventional doses used in the treatment of idiopathic PUD/GERD or they are frequently used for UGI symptoms in non-ZES patients [9,59,88,92,217,218,219,220,221]. In contrast to PPIs, with longterm treatment with H_2_R antagonists in ZES patients, higher dosing and/or more frequent dosing, are almost always needed than they are generally used to treat the typical patient with idiopathic GERD/PUD [33,59,79,80,217,222,223], thus helping to raise the suspicion that the patient may have ZES as a possible diagnosis. Hence, in the past when ZES patients were treated with the conventional doses of H_2_R antagonists which were widely used in non-ZES patients, they continued to have symptoms, raising the possible awareness that they may have ZES. However, this very important clinical observation that suggested the correct diagnosis of ZES is not with PPIs treatment [33,200,224]. The result of this combination of differences is that the use of PPIs greatly complicates the diagnosis of ZES [185].

#### 2.2.2. Current ZES Controversy in Diagnosis

##### Background to Current Controversy in ZES Diagnosis

Similarly to the diagnosis of any F-pNET syndrome, the diagnosis of ZES requires the demonstration of the unphysiological release of the causative hormone [200], which in the case of ZES requires the demonstration of an inappropriate fasting serum gastrin (FSG) level for the degree of acid secretion. This historically required a measure of the FSG level and a measurement of acid secretion (Table 1).

The classical approach to the diagnosis and criteria for the diagnosis of ZES widely used until the recent changes discussed above occurred (i.e., lack of availability of acid measurements, introduction of PPIs) are listed in Table 1 (Part A). These classical criteria have been well validated. For example, in eighty-one consecutive patients diagnosed with ZES at the NIH using these classical criteria, who subsequently had surgical explorations, a gastrinoma was found in all [55] and in forty-eight cases of sporadic ZES diagnosed using similar classical criteria, a primary or metastatic gastrinoma was found in all patients at surgery [250].

Almost invariably, the first study performed when the diagnosis of ZES is suspected is a FSG assay [203,226]. FSG levels are elevated in almost all patients with ZES (>99%), except in some rare circumstances, such as post-parathyroidectomy in MEN1/ZES or post-noncurative gastrinoma resection [251,252,253,254,255]. Unfortunately, while the FSG level has a very high sensitivity for diagnosing ZES it has low specificity, because no matter how high the FSG level is, it alone is not sufficient to establish a definitive ZES diagnosis, with very elevated values (>5–10× increased) reported in a significant percentage of patients with chronic atrophic gastritis and even in some patients without ZES taking PPIs [206,207,208,209,210,211,212,213,253,256,257,258,259]. This low specificity of increased FSG levels for ZES occurs because the presence of fasting hypergastrinemia can either be a result of a physiological response which develops in response to any condition that causes chronic hypo-/achlorhydria or it may be a pathological or inappropriate response, which can occur in a few disorders which are alos associated with either the presence of normal or even elevated gastric acid secretion. In humans the principal disorders causing physiological hypergastrinemia are chronic atrophic gastritis [CAG]/pernicious anemia, the use of PPIs, or the presence of H. pylori infections, which occur in patients without ZES with GERD/PUD symtoms much more frequently than ZES [260,261,262,263,264], and thus need to be excluded as a cause of the hypergastrinemia to establish a firm diagnosis of ZES. Furthermore, there are other disorders besides ZES, which can be associated with fasting hypergastrinemia and an acid secretion more elevated than ZES, and thus need to be excluded, including such disorders as H. pylori infections (frequent), and other infrequent disorders such as rare, chronic renal failure, antral G cell hyperfunction/hyperplasia, gastric outlet obstruction, short bowel syndrome, and retained gastric antrum syndrome [227,265].

Because of the low specificity of FSG values alone for the diagnosis of ZES, the second study typically recommended to establish the diagnosis of ZES was an assessment of gastric acid secretory rates or gastric pH [33,155,202,227,266] (Table 1 (Part A)). These FSG and acid secretory criteria combination criteria summarized in Table 1 (Part A) became the established diagnostic criteria for ZES recommended by all societies and experts, including the American NANET’s and European ENET’s guidelines, as well as recommendation by numerous experts for the diagnosis of ZES [34,54,59,206,267,268,269]. Furthermore, it was recommended to establish both criteria; a potent acid secretory drug such as a PPI had to be stopped for up to one week and then determine the gastric pH and FSG, which allowed the PPI-induced hypergastrinemia to be reversed, and gastric acid pH/output to be assessed [34,59,206,267,268,269]. As partially discussed above, there were two problems with this approach: first, gastric acid output measurements are no longer available in most hospitals, and the second, a possible safety problem with this approach, if the patient actually has ZES. There have been a number of reports of severe gastric peptic/GERD complications in ZES patients after abruptly stopping PPIs [32,270,271]. Although a number of approaches have been proposed to avoid these complications during PPI withdrawal such as tapering the PPI dose slowly or only partially, or substituting H_2_Rs [200,271], these are uncommonly used, except in some specialty centers. Lastly, to distinguish ZES from other disorders causing modest fasting hypergastrinemia (<10-fold increase over normal) and hyperchlorhydria, the use of secretin testing was typically recommended (Table 1 (Part A)) [76,229,270,272,273,274,275]. The secretin test was recommended because of its convenience, sensitivity, specificity, and lack of side effects [28,229,276]. An NIH study of a large number of NIH ZES patients and ZES patients undergoing secretin testing in the literature (i.e., >500) [229] reported that the best criterion for a positive test in ZES patients was an increase in serum gastrin with secretin ≥ 120 pg/mL, which had a sensitivity of 94% and specificity of 100% for identifying ZES patients [229]. These criteria had a greater sensitivity than previously proposed criteria of increases of ≥200 pg/mL, ≥50% over basal or ≥110 pg/mL [228,277,278,279], and therefore is the criterion that was generally recommended [54,272,280]. Unfortunately, in many centers/hospitals, secretin is not readily available and so, at present, it is uncommonly used. The result of the unavailability of gastric acid testing, the potential dangers of PPI withdrawal in a true ZES patient, and lack of widespread availability of secretin testing has led to the current controversy of what method should be used to diagnose ZES at present. The classical criteria are now used only in a few referral centers, and as shown in (Table 1 (Parts A and B)) of 35 recent case reports of ZES in the literature from the years 2013 to 2024 [28] (Table 1) in <5% of the cases the classical diagnostic ZES criteria was used to diagnose the case of ZES reported, largely due to the failure to measure gastric output/pH. As seen in (Table 1 (Part B)) these thirty-five recent case reports of ZES patients were first suspected on the basis of their clinical history of suggesting refractory PUD/GERD or the presence of its complication [230], with subsequent demonstration of hypergastrinemia. In some cases, there is further conformation such as a positive conventional imaging study (CT, MRI) showing an abdominal/pancreatic mass or a positive somatostatin receptor imaging (SRI) study (^68^Ga-DOTATATE PET/CT or ^111^In-DTPA-ocreotide with SPECT/CT imaging or less frequently a percutaneous biopsy (liver, etc.) showing gastrinoma.

Recently there have been four papers using or proposing different criteria for the diagnosis of ZES or ZES/MEN1 from four different centers who have extensive experience with this disease [16,28,201]. **The first study** [16] is ENETs guidance paper providing advice for clinicians on the diagnosis and treatment of F-pNET syndromes including gastrinoma/ZES. In this paper [16], Query 2/Figure 2 specifically deals with the question of what biochemical tests should be performed in a patient with a clinical suspicion of ZES (gastrinoma). After suspecting ZES based on the clinical history, they recommend [16] as the primary diagnostic approach to establish the diagnosis of gastrinoma—one must demonstrate an FSG level > 10× ULN and a gastric fluid pH ≤ 2 in the absence of PPI use (classical definition = Table 1 (Part A)). In clinically suspected patients with FSG increased 1–10× URL, it is recommended a UGI endoscopy with an endoscopic ultrasound evaluation (EUS) be performed, as well as a CT or MRI examination and an SRI examination. As an alternative diagnosis approach [16] for diagnosis of gastrinoma it was recommended that a positive diagnosis could be made by using a combination of suggested clinical symptoms and elevated FSG level with the presence of a duodenal/pancreatic NET with gastrin expression by immunohistochemistry or the presence of positive uptake on somatostatin receptor imaging (SRI). **A second study** [201] dealt with the diagnosis of ZES in MEN1 patients (ZES/MEN1), which comprises 20–30% of all ZES cases [49,281,282,283,284,285,286,287,288,289] and is the most frequent F-pNET syndrome seen in MEN1 patients occurring in 20–61% (mean-54% [9 series]) [25,51,232,290]. The recommendations were based on responses of 59 ENETs centers of Excellence [201], which reported that the biochemical modalities used for gastrinoma diagnosis in the various center included 98% using FSG, 36% using FSG only, 55% using FSG plus gastric pH, FSG plus secretin or calcium stimulation test (7%), and secretin stimulation test only (2%). These results show the marked heterogeneity and lack of complete agreement on which studies should be uniformly used for ZES/MEN1 diagnosis, even in highly specialized centers. **A third study** [25] including criteria for diagnosis of ZES in 63 MEN1 patients (ZES/MEN1) used the following criteria for diagnosis of ZES: pathology only (11%), biochemical and pathology together (24%), biochemical only (64%), and increased FSG with imaging (2%), and the basis of the biochemical diagnosis was >10× ULN for FSG (71%) when the PPI was not stopped, >2× increase FSG without PPI, or >5× ULN with PPI, and in 16% FSG the above criteria was not fulfilled. No measure of gastric acid output or pH was used in this study. **A fourth recent study** [203] examined the controversies in diagnosing ZES at present, in detail, and for the first time provided both the rationale for and proposed possible new criteria which could be used, which would support the diagnosis of ZES, that did not involve the assessment of gastric pH.

In this study [203], the authors proposed possible new criteria for diagnosing ZES in patients with fasting hypergastrinemia in the absence of PPI therapy [gastric pH data not available]. These criteria [203] were divided into those strongly supportive of ZES diagnosis (clinical; PUD with positive SRS; positive histology; positive secretin test; PUD with MEN1; gastrinomas by histology); moderately supportive of ZES diagnosis (positive SRS with positive histology with absence of atrophic gastritis [291,292,293,294]); weakly supportive of ZES diagnosis (MEN1 present with positive SRS) [291,292,295,296,297]. This study [203] also proposed possible new criteria supporting the diagnosis of ZES in patients with fasting hypergastrinemia taking PPIs [gastric pH data not available]. These criteria were divided into those moderately supportive of ZES diagnosis (patient with active PUD, strong history of PUD, or improvement in diarrhea with PPI treatment with positive histology for NET); weakly supportive of ZES diagnosis [203,293,294] or minimally supportive of ZES diagnosis [232].

The authors of the above study point out that although many current reports involving the diagnosis of ZES (Table 1) used the criteria for diagnosis of an elevated FSG combined with a positive SRI study [203], this is not specific for ZES. Its low specificity occurs because many patients with achlorhydric/hypochlorhydria frequently have a non-gastrinoma NET that results in a positive SLI imaging and thus, even though diagnosed as ZES with these criteria, they do not actually have the disease. This may be particularly true in MEN1 patients suspected of having ZES while taking a PPI, because these patients develop numerous neuroendocrine tumors in numerous locations, which can be positive on SRI, but which are not gastrinomas. Furthermore, some authors propose that gastrin provocative testing performed while the patient is taking PPIs can be substituted for the conventional criteria involving stopping the PPI to assess gastric pH [203]. Unfortunately, most studies [298,299], but not all [300], have concluded that this approach is not a reliable alternative, because a number of provided evidence that secretin test results are not reliable in patients taking PPIs [298,299]. However, a recent study [300] has challenged this generally held conclusion, because in twenty-eight ZES patients being treated with PPIs, the authors found that no false positive or false negative secretin tests occurred with PPI treatment. The authors in the fourth recent study reviewed above, which has new proposed possible criteria that could assist in ZES diagnosis without measuring gastric pH [203], point out that these new proposed criteria [203] only support the diagnosis of ZES. These proposed criteria have not been prospectively evaluated and are not as strong as the classical criteria using the combination of an increased FSG and with a gastric pH ≤ 2. The only reason that these possible new criteria were proposed for possible study is because >95% of physicians are not using the established criteria for the diagnosis of ZES [Table 1 (Part A)], and it is not apparent this practice will be reversed in the future. None of the above recently proposed criteria for diagnosis that do not involve the classical assessment of FSG combined with a gastric fluid pH/acid output assessment can be considered to unequivocally establish the diagnosis of ZES, as did the classical criteria, which are now rarely used; many are not applicable to all patients suspected for the presence of ZES. It is unclear that these will be generally used; to establish these, some prospective evaluation of them is needed. Because the diagnosis of ZES has such an important consequence for the patients including both immediate and lifelong treatment requirements [203,225], it is imperative that the diagnosis be firmly established. The best method to ensure this, at present, is to refer these patients to a center that has expertise in the diagnosis of ZES to firmly establish the diagnosis by the established classical criteria.

Serum chromogranin A (CgA) has been proposed as a useful marker for NETs/pNETs [259,301,302,303,304,305,306]. Serum CgA is a sensitive marker in patients with ZES [9,259,304,307,308,309,310,311]; however, numerous studies have shown its usefulness is greatly limited in ZES patients, similarly to other patients with NETs/pNETs, because of its low specificity [305,306,307,312]. Numerous non-ZES/NET conditions can result in an elevated CgA decreasing its specificity [305,306,312]. Furthermore, its low specificity is particularly a problem with suspected ZES patients because of the presence of tumor-induced hypergastrinemia causing gastric ECL hyperplasia in the actual ZES patients, resulting in an increased CgA level not being an expression of only a possible gastrinoma as well as the fact that the use of PPIs also resulting in ECL hyperplasia in non-ZES patients results in a possible false positive ZES/pNET/NET diagnosis. These results can complicate the interpretation of CgA resulting in reduced specificity in patients suspected of having ZES [305,306,307,312].

### 2.3. Current ZES Controversies in Nonsurgical Aspects of ZES/MEN1 Management

#### 2.3.1. Background to ZES Controversies in Nonsurgical Aspects of ZES/MEN1 Management

The most controversial point in MEN1/ZES patients is the role of surgery directed against the gastrinoma which will not be discussed in this paper dealing with medical controversies but instead dealt with in a later paper on controversies in surgical ZES issues. Instead, in this paper three controversial/unclear areas of nonsurgical management will be briefly discussed. These include in MEN1/ZES patients the management of gastric carcinoids (Type 2 carcinoids); controversies of the roles of imaging/tumor localization for gastrinomas/pNETs in the initial/follow-up management of MEN1/ZES patients; and the possible important prognostic value of type of MEN1 germline mutation present in MEN1/ZES patients.

ZES patients with MEN1, which comprise 20–30% of all ZES cases [49,281,282,283,284,285,286,287,288,289], have a number of unique concerns not seen in patients with sporadic ZES, of which a number are controversial or have not been systematically studied and thus the management is unclear or controversial. ZES/MEN1 patients differ from patients with sporadic ZES in a number of features related both to the gastrinoma itself and also to the presence of other NETs/other tumors in other organs which in some cases can cause functional syndromes, such as hyperparathyroidism, which can affect the management of the ZES [35,251,281,313,314,315]. Specifically, MEN1 is an autosomal dominant disorder due to mutations in the MEN1 gene on the long arm of chromosome 11 (11q13). The MEN1 gene has ten-exons encoding for a 610 amino acid protein, MENIN [290,316,317,318,319]. The exact molecular alteration that occurs with MENIN mutations that results in pNETs, including gastrinomas, is not clear. However, it is known that MENIN is a nuclear protein that interacts with a large number of proteins [50,290,320,321,322,323,324], including SMAD3; Jun D; FANCD2, nucleoside diphosphate kinase, NM23 cytoskeletal-associated proteins, and various histone-modifying enzymes [50,317,320,321,323,324]. The MEN1 mutations which occur in MEN1/ZES patients result in the development of NETs and hyperplasia in multiple endocrine organs. Typically, patients with MEN1 were reported to develop hyperparathyroidism; pancreatic NETs (NF-pNEN > gastrinoma > insulinoma >> other); and pituitary adenomas [50,281,290,325,326]. Each of these different neuroendocrine tumors could be associated with or without a functional syndrome [50,281,290,325,326]. Historically, the vast majority of MEN1 patients have hyperparathyroidism at diagnosis. Nonfunctional pNETs (NF-pNET) occur in 80–100% patients with the majority developing microscopic NF-pNET. As a result NF-pNET only are associated with symptoms in 0–13% of MEN1 patients [281,290]. The most frequent F-pNET seen in MEN1 patients are gastrinomas which are reported in a mean frequency of 54% with a range of 20–61% in various studies [50,232,290,326,327]. In addition to the classical triad of NET/endocrine tumors MEN1 patients develop, recent studies report NETs/endocrine tumors in a number of other locations, as well as nonendocrine tumors in a number of locations. Adrenal tumors, which are primarily nonfunctional, and thyroid tumors can occur in <50%, and MEN1 patients have an increased incidence of carcinoids (stomach, lung, thymus) [50,326,328]. Numerous more recent studies report that MEN1 patients also develop smooth muscle tumors (leiomyomas, leiomyosarcomas), CNS tumors (meningiomas, schwannomas, ependymomas), skin tumors (angiofibromas > collagenomas > lipomas > melanoma), as well as breast, urological, and other tumors [50,290,326,328,329,330,331,332,333].

The presence of MEN1 in ZES patients has important effects on the pathology of the gastrinoma and its behavior, the prognosis of the patient, and all aspects of the management of the patient, including the need for genetic counseling. In MEN1/ZES patients the gastrinomas are similar to those in sporadic ZES patients in that they are primarily duodenal in location and frequently associated with lymph node metastases; however, they differ in MEN1/ZES from sporadic ZES in that the MEN1 duodenal gastrinomas are almost variably multicentric, smaller, and multiple [334,335,336,337,338], as well as the fact they differ in biological behavior in that the rate of liver metastases is significantly higher in sporadic cases than in MEN1/ZES patients [337]. In addition, the distribution of the tumor grades in patients with gastrinomas with MEN1/ZES differed from those with sporadic ZES in having more patients with lower grades (G1: 83% vs. 39%; G2 (11% vs. 54%) G3: (5.6% vs. 6.1%) [337]. This increased proportion of patients with gastrinomas having aggressive behavior with higher grades of the primary gastrinoma in sporadic ZES patients correlates with results from prospective NIH clinical studies of patients with either sporadic ZES or ZES with MEN1. In the study of patients with sporadic ZES, 25% had gastrinomas which demonstrated aggressive growth behavior during the study period [5], differing from patients with MEN1/ZES, with only 14% demonstrating aggressive growth during the study period [339]. These results are consistent with a number of other studies in ZES patients with or without MEN1 which report that MEN1/ZES patients have a better prognosis than patients with sporadic ZES [5]. Patients with ZES can primarily have a duodenal primary tumor; however, gastrinomas also occur in the pancreas, particularly in sporadic cases. Duodenal gastrinomas in MEN1/ZES patients have a different pathogenesis than sporadic duodenal gastrinomas. In MEN1/ZES patients, duodenal gastrinomas have been shown to arise from the duodenal G cells by a process of progressive hyperplasia in a similar manner to that proposed for the response of ECL cells to gastrin in the stomach [340,341]. In MEN1/ZES patients, studies propose that a key mutation event that contributes to the subsequent development of the multifocal duodenal gastrin neoplasms is the allelic deletion of the second MEN1 allele [340,342]. However, this sequence was not seen in sporadic duodenal gastrinomas [340,342]. The presence of the other non-gastrinoma NETs in MEN1/ZES can also affect gastrinoma behavior as well as management of the gastrinoma. The concomitant presence of hyperparathyroidism, which is present in >95% of MEN1/ZES patients due to the parathyroid hyperplasia characteristically seen in MEN1 patients [5,314,343], can directly affect the behavior of the gastrinoma by stimulating the release of gastrin, increasing the basal acid output, affecting gastrin provocative secretin test positivity used in ZES diagnosis, and by increasing the difficulty in suppressing the acid secretion by medical therapy [33,251,254,315].

#### 2.3.2. Nonsurgical MEN1/ZES Controversies

##### Nonsurgical MEN1/ZES Controversies: Management of Gastric Carcinoids (Type 2) in MEN1/ZES Patients

MEN1/ZES patients have a much higher rate of developing gastric carcinoid tumors than patients with sporadic ZES [i.e., 23–70% vs. <1% [93,344,345,346,347,348,349]. Based on the results of two NIH prospective studies it has been estimated gastric carcinoids occur at least with seventy-fold greater frequency in MEN1/ZES-patients [344,345]. This estimate was calculated from the differences in the NIH prospective study of 57 consecutive MEN1/ZES patients in which gastric carcinoid tumors were found in 23% of the patients [344], whereas in a similar study involving 106 patients with sporadic ZES [345], 0% had gastric carcinoids. Gastric carcinoid tumors in MEN1/ZES are classified as a Type 2 carcinoid tumor, making up 5–6% of all gastric carcinoids and are similar to Type 1 gastric carcinoids which develop in patients with chronic atrophic gastritis/pernicious anemia (70–80% of all gastric carcinoids), in that both are associated with the presence of chronic hypergastrinemia, but Type 1 gastric carcinoids are seldom malignant, whereas Type 2 gastric carcinoids may be malignant with nodal metastases. Type 3 gastric carcinoids (6–8% of all gastric carcinoids) are not associated with hypergastrinemia [348,350,351,352,353,354,355]. Based primarily on series involving retrospective reviews of cases either from the literature or from various centers, Type 2 gastric carcinoids are reported to be primarily small in size and frequently multiple involving most of the stomach; show well-differentiated GI pathology; metastasize in 10–30% which is higher than the 2–9% seen in Type 1 and 80–100% seen in Type 3 gastric carcinoids; and be a cause of death in <10% of patients, as opposed to >50% in Type 3 and 0% in Type 1 carcinoids [348,350,351,352,353,354,355,356]. The management of Type 2 gastric carcinoids has not been well studied prospectively and has a number of aspects which are unclear and will be discussed further in the paragraphs below.

**Figure 6:**
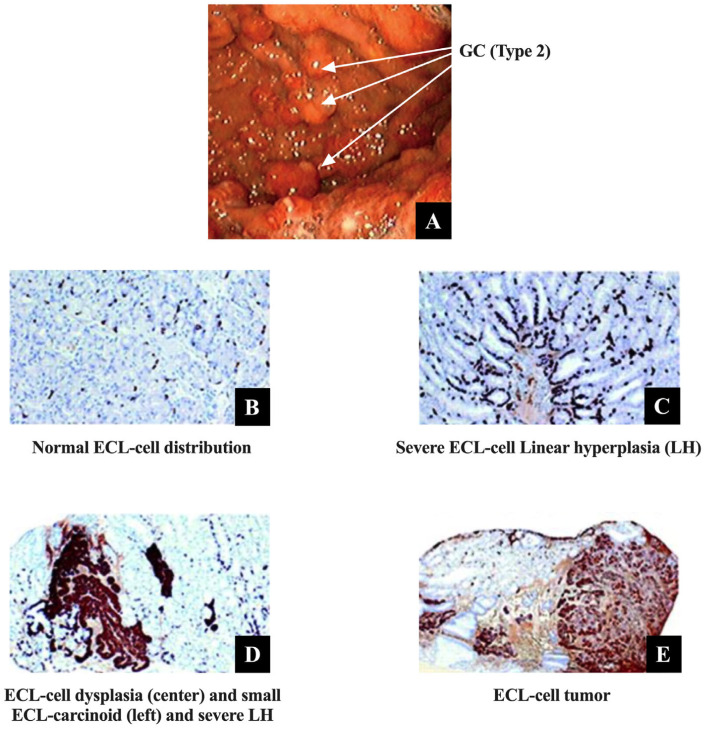
Gastric carcinoids type 2 in MEN1/ZES and their precursor lesions. Panel (**A**) shows an upper gastrointestinal endoscopy result from a patient with longstanding MEN1/ZES demonstrating extensive development of multiple gastric carcinoids (arrows). Multiple gastric carcinoids occur in 3–21% of patients with MEN1/ZES but are rare in patients with MEN1 without ZES. In contrast to Type 1 gastric carcinoids seen in patients with pernicious anemia/chronic atrophic gastritis, Type 2 gastric carcinoids can be malignant in 10–30% which is higher than the 2–9% seen in Type 1. Results are modified from [350]. In Panels (**B**–**E**) we showed the proposed sequence of increasing ECL-cell proliferative changes that have been proposed for the development of Type 2 gastric carcinoids due to chronic hypergastrinemia. Gastric biopsy specimens results from different MEN1/ZES patients showing different degrees of ECL-cell changes on chromogranin A staining are shown. In Panel (**B**) we showed a qualitatively normal distribution of chromogranin A-positive immunoreactive cells (in *brown*) in the oxyntic mucosa; Panel (**C**) shows severe linear hyperplasia (LH) of ECL-cells (in *brown*); Panel (**D**) shows a small intramucosal ECL-cell carcinoid tumor (0.9 mm on the *left*) associated with an ECL-cell dysplastic lesion (in the *center*) and severe LH of ECL-cells (on the *right*); and finally, Panel (**E**) shows an ECL-cell carcinoid tumor from a mucosal nodule. Results are modified form [344] and are freely available as author RTJ is from US government agency NIH.

Type 2 gastric carcinoids develop at an increased rate in MEN1/ZES patients because of the presence of chronic hypergastrinemia combined with the molecular effects of the presence of the MEN1 gene mutations (Figure 6A).

Chronic hypergastrinemia stimulates proliferation of the gastric enterochromaffin-like cells (ECL-cells), which show such a response in almost all ZES patients, but is especially accelerated in patients with MEN1/ZES [93,344,345,347,357,358,359]. Numerous studies report that in ZES patients the number of gastric ECL cells is increased approximately two-fold [93,155,357,360]. In ZES patients the gastric ECLs show a spectrum of proliferative changes similar to those described in various animal hypergastrinemic models and in some cases not only can these result in dysplastic changes but also neoplastic changes [155,344,345,361,362]. From these various studies of the changes in gastric ECL cells in animals and humans with chronic hypergastrinemic states, a model sequence of progressive changes has been proposed to occur in the gastric ECL cells with chronic hypergastrinemia, with the ECL cells undergoing a progressive hyperplasia changes leading to neoplasia. This begins with the development of simple hyperplasia, followed by progressive degrees with simple hyperplasia progressing to linear hyperplasia, followed by the development of micronodular hyperplasia, adenomatoid hyperplasia, dysplasia (pre-carcinoid), leading to the final development of carcinoids [155,362,363,364] (Figure 6B–E). In two prospective NIH studies of gastric ECL changes in ZES patients with or without MEN1, greater than 98% of ZES patients demonstrated ECL hyperplasia [344,345]. In the study of ZES patients with sporadic ZES, 50% of patients were found to have advanced ECL changes with 7% having dysplasia [345] and in the patients with MEN1/ZES, 53% had advanced ECL changes [344] (Figure 6B–E). Similarly in other studies, 75–100% of the ZES patients had some degree of ECL hyperplasia [258,347,359]. In ZES, there is a close correlation between the degree of ECL hyperplasia and the fasting serum gastrin level [155,344,345].

Proliferative effects on gastric ECL cells are present in almost all MEN1/ZES patients because chronic hypergastrinemia exists at some point in all patients with MEN1/ZES, and it is lifelong in 90–100%. Lifelong hypergastrinemia occurs because the role of surgical resection of the gastrinoma remains controversial in these patients and many patients with small pNETs are being initially treated by a watch-and-wait approach rather than surgery resulting in sustained, lifelong hypergastrinemia [26,27,41,45,46,365,366,367,368,369], which is due to the low surgical cure without aggressive resections, such as Whipple resections, which are not routinely recommended [27,45,46,370]. Thus, very few patients with MEN/ZES are rendered disease free surgically. The low cure rate in most patients with MEN1/ZES with standard operations which lead to this watch-and-wait approach occurs for a number of reasons. MEN1/ZES patients almost invariably have multiple duodenal gastrinomas which are microscopic to small (many <0.5 cm) and thus difficult to find at surgery and completely remove even with a duodenectomy. In addition, >50% have metastatic lymph nodes at the time of surgery [41,334,371], making it further difficult to cure the patient. While aggressive resections such as Whipple could cure the patient, because of their side-effects they are not routinely recommend [27,45,46,370]. This approach was further supported by the fact that medical control of acid hypersecretion can be very effective if properly performed [8,13,15,33,59], the long-term nonsurgical outlook is very good in patients with small pNETs, all contributing to the low cure rate and nonsurgical approach which is frequently adopted in MEN1/ZES patients with small pNETs on imaging studies (<1–2 cm on imaging). The excellent long-term surgical outlook in MEN1/ZES patients with small pNETs has been demonstrated in numerous studies reporting that if the preoperative imaging studies in an MEN1/ZES patient identify a pancreatic-duodenal tumor <1–2 cm in diameter (i.e., which are usually not the primary duodenal gastrinoma, but an adjacent positive metastatic lymph node or an NF-pancreatic pNET [336,372]), these patients have an excellent long-term prognosis; in fact, survival is not different from MEN1 patients without pNEN seen in some studies [373,374].

A number of factors have contributed to the current situation of the lack of detailed information allowing the best proven approach to be clearly defined for the management of Type 2 gastric carcinoids in MEN1/ZES [348,351,353,375,376,377]. Type 2 gastric carcinoids are the least common of the three classes of the gastric carcinoids, so they are relatively infrequent [348,350,351,352,353,354,356]. Furthermore, until the introduction of PPIs in the late 1980s, many MEN1/ZES patients underwent a total gastrectomy to control their severe hyperchlorhydria, because it was the only form of treatment that was successful in all cases [3,47,378,379,380,381]. Furthermore, many centers were unable to medically control the hypersecretion in these patients, which is more difficult to control than in sporadic ZES cases using H_2_Rs [5,382,383]; primarily, high, frequent H_2_R doses were required by most MEN1/ZES, which need to be individually titrated [223] and was not frequently available. The result of this is that except for the ZES/MEN1 patients at NIH and a few other specialties centers whose acid hypersecretion, in all, was controlled both acutely and long-term by medical treatment [33], there were few MEN1/ZES patients with intact stomachs that could be studied for possible Type 2 gastric carcinoid development. The result of this low frequency of cases is that at present there are only a few reports of patients with small numbers of cases with MEN1/ZES with Type 2 gastric carcinoids who have been followed prospectively and thus their true natural history is largely unknown [349,384]. In the early retrospective reports of combined series of Type 2 gastric carcinoids it was usually considered that Type 2 carcinoids were generally benign with minimal life-threatening risk, similar to Type 1 carcinoids [348,349,384,385,386]. However, a few studies involving a small number of Type 2 MEN1/ZES patients have reported either aggressive growth of the gastric carcinoid and even a fatal outcome in some patients [351,375,386,387,388,389]. It is therefore increasingly recognized that Type 2 gastric carcinoids are more aggressive than Type 1 gastric carcinoids; however, this has not resulted in marked changes in most guidelines in the proposed treatment approaches to patients with these two different carcinoid groups. In both cases, the primary recommend approach is endoscopic surveillance and resection of visible lesions [350,355,390]; however, others [354] have recommended curative resection of the gastrinoma as a primary approach, which, as discussed above, unfortunately, is rare (<5%), with the currently recommended surgical approaches. In many MEN1/ZES patients with long-standing ZES with Type 2 gastric carcinoids, the entire corporal area of the stomach can be covered by gastric carcinoids of varying sizes (Figure 6A) and thus it may not be practical or possible to remove all endoscopically; furthermore, they may recur. For this reason, some have proposed the use of long-acting somatostatin analogs in patients with gastric carcinoid Type 1/2 [391,392,393,394,395,396] as well as the possible use of the CCK_B_ receptor antagonist, netazapide [397,398] in patients with multiple or extensive gastric carcinoids, such as seen in MEN1/ZES patients. At present, how the follow-up of MEN1/ZES patients with multiple gastric carcinoids should be modified is unclear and will remain so until systematic studies define the natural history of these and their long-term malignant potential. This will be somewhat complicated by the fact that liver metastases and gastrointestinal lymph node metastases in patients with long-standing MEN1/ZES can come from multiple sources (duodenal, pancreatic, gastric, thymic, lung) and a given patient can have simultaneous metastases from multiple sources [399], so these will have to be distinguished to establish the natural history of each primary source to allow therapy to be appropriately adjusted.

A recent study [313] provides some results that may provide important insights into further approaches to patients ZES/MEN1 patients for managing the possible development of Type 2 gastric carcinoids. In this study, sixteen patients with ZES/MEN1 who were diagnosed and their gastric findings were analyzed. A total of 62% had gastric ECL hyperplasia which was mild in most patients, 12% had small gastric carcinoid tumors [313], with increases in serum gastrin to only 206 pg/mL These percentages are significantly less frequent than reported in the NIH prospective study of MEN1/ZES patients [344] and less than reported in a number of older studies [258,347,359]. These less severe gastric changes are likely due to a combination of factors including the earlier and aggressive use of Whipple resections and other gastrinoma resections in these patients, resulting in significantly less severe hypergastrinemia than in previous studies; shorter periods of sustained advanced hypergastrinemia and shorter periods of follow-up prior to the study existed on only medical antisecretory treatment. Nevertheless, these results suggest aggressive treatment of the gastrinoma in these patients may ameliorate the development of advanced gastric mucosal changes.

##### Nonsurgical MEN1/ZES Controversies: Controversy of Whether Genotype–Phenotype Correlations Exist in MEN1 Patients Including MEN1/ZES Patients

- Background to Controversy in Phenotype/Genotype Correlations Occurring in MEN1 Patients and MEN1/ZES Patients

As discussed in detail in the preceding paragraphs, MEN1 is an autosomal dominant disorder due to mutations in the MEN1 gene on the long arm of chromosome 11 (11q13). The MEN1 gene has ten-exons encoding for a 610 amino acid protein, MENIN [316,317,318,319,400]. The exact mechanism leading to the development of the various NETs, pNETs, and other endocrine/nonendocrine abnormalities in MEN1 or MEN1/ZES patients remain unclear. However, it is now well established that MENIN is a nuclear protein that interacts with a scaffolding protein, interacting with number of other proteins, particularly involved in controlling cell growth, gene expression, gene transcription, cell signaling, and regulation of the integrity of the genome [50,320,321,322,323,401]. In various studies 90–95% of patients fitting the classical clinical criteria for diagnosis of MEN1 [326] are found to have germline MEN1 gene mutations, whereas the remaining 5–10% are not found to have a MEN1 gene mutation [316,317,321,400,402,403,404]. There have been >1300 different mutations in the MEN1 gene described in MEN1 patients [223,400]. These mutations have been overall reported in 23% to be a nonsense mutation; 41% a frameshift mutation with 68% due to a deletion; 32% to an insertions; 6% an in-frame deletion or insertion; 9% a splice site variation; 20% a missense mutation; and 1% a large deletion, which in 75% of patients results in functioning as an inactivating mutation [316,400,402,403,404,405].

- Controversy in Phenotype/Genotype Correlations Occurring in MEN1 Patients and MEN1/ZES Patients

Numerous studies have reported the results of investigations into whether MEN1 genotype–phenotype correlations exist, and results are briefly summarized in Table 2.

In contrast to MEN2 [424,425] and a number of other inherited disorders, in the case of MEN1, most but not all studies (Table 2) report no genotype–phenotype correlation. This includes no genotype–phenotype correlation between the MEN1 gene mutation and generally with the various types of other endocrine/nonendocrine abnormalities MEN1 patients develop. This concludes for the endocrine MEN1 disorders no generally recognized genotype–phenotype correlation for parathyroid-hyperplasia resulting hyperparathyroidism, pituitary adenomas, and functional hypersecretory pituitary syndromes, adrenal adenomas/disorders, pNETs, and functional syndromes; or the development of carcinoids (gastric, thymic, lung) as well as for the development of nonendocrine tumors/abnormalities (skin-[angiofibromas, collagenomas], CNS tumors [meningiomas, schwannomas, ependymomas, smooth muscle tumors, breast cancer]) [290,317,328,331,400,426]. This also includes the general lack of a genotype–phenotype correlation for the natural history of the patients in terms of aggressiveness of a given tumor type, or correlation with the outcomes of various therapeutic approaches including surgery.

However, as evident from some reports in 2, a number of studies show positive genotype phenotype correlations as well as correlations between genotype and prognosis. Numerous studies report that MEN1 patients with or without a gastrinomas have a shortened survival compared to the general population, which is now primarily due to MEN1 related deaths from various malignant NETs, with deaths due to pancreatico-duodenal NETs being the most prominent [25,284,328,420,427,428,429,430,431,432,433,434,435,436,437,438,439,440,441,442,443,444,445,446,447]. In addition to these studies showing positive genotype phenotype correlations in MEN1, a number of studies have reported possible genotype phenotype correlations in case reports as well as from studies of members of large kindreds with the same genotypic mutation which have differing clinical courses/phenotype expression [318,408,448,449,450].

Natural history studies of MEN1/ZES patients show that in 14% of the patients, the pancreatic-duodenal NETs pursue an aggressive course [328,339]. Although other studies report that MEN1/ZES patients have a more benign course and better prognosis than patients with sporadic ZES [339,451,452,453], the above studies show their survival is still significantly shortened. Therefore, the identification of any genotype phenotype correlation could be of significant value, especially in tailoring the long-term evaluation of these patients for the development of new NET disorders or for the course of a given one, knowledge will relate to the timing of surgery and repeat screening. At present, the significance of the positive genotype–phenotype correlations shown in Table 2 or the genotype–phenotype relations from studies of large families are unclear. For example, a number of studies in Table 2 report exon 2 mutations, particularly frameshift/nonsense mutations or those affecting JunD interactions [53,405,419,420] or the presence of a frameshift/nonsense, truncating mutation [408,411,418,423], were associated with more frequent development or malignant behavior of various pNETs, with decreased survival or more aggressive behavior. In contrast, other studies [328,408,419] report association with one or both of these mutations was associated with less advanced disease and better survival. The true significance of these proposed genotype–phenotype correlations needs to be investigated by perspective, long-term studies. Also, insights into the importance of these genotype–phenotype mutations may be provided by comparative studies of patients with clinically diagnosed MEN1 due to the presence of a MEN1 mutations (80–95%) compared to patients without MEN1 mutations but have clinically diagnosed MEN1 (5–15%). A small number of such studies have been performed and show that, in general, the clinical course of these two forms of MEN1 differ with MEN1 patients without MEN1 mutation having a milder disease, later onset, lack of recurrent hyperparathyroidism, lower incidence of entero-pancreatic tumors or skin tumors (angiofibromas, collagenomas) [418,422,450,454,455,456]. Furthermore, in 31–58% of patients with sporadic gastrinomas [457,458] without a germline MEN1 mutation present, there is an MEN1 gene mutation in the gastrinomas, which is a similar situation in other sporadic non-gastrinoma pNETS which can have MEN1 mutation only in the pNET in 20–40% [459,460,461]. A comparison of these two groups of patients and the clinical course of the pNETs in each may also provide important genotype–phenotype insight into the effect of various MEN1 mutations on the behavior of a given pNET type.

##### Nonsurgical MEN1/ZES Controversies: Controversies of the Roles of Imaging/Tumor Localization in MEN1 Patients for Gastrinomas/pNETs in Their Initial/Follow-Up Management

- Controversy of the Role of Specific NET Imaging Studies in NET/pNET Diagnosis in/ZES-MEN1/MEN1 Patients. Background to Controversies of the Roles of Imaging/Tumor Localization in MEN1 Patients for Gastrinomas/pNETs in Their Initial/Follow-Up Management

Similarly to patients with sporadic NETs/ZES/pNETs, patients with MEN1-ZES/MEN1 require tumor localization studies in all phases of their management, including in some cases for diagnosis; to assess the role of surgery whether for possible cure or cytoreduction; to assess the need for anti-tumor therapy or the response to anti-tumor therapy; and to assess tumor recurrence [8,16,58,118,201,326,462,463,464,465,466,467,468,469]. However, patients with ZES/MEN1, similarly to other MEN1 patients, have a number of special features that affect the management and, in particular, various aspects of tumor localization which in some cases are controversial. These controversies will be discussed in more detail in the next paragraph, but they include the role of specific NET imaging studies in NET/pNET diagnosis in/ZES-MEN1/MEN1 patients; which tumor localization studies should be performed both initially and during follow-up studies; and when the tumor localization studies should be performed during follow-up and with what frequency [57,58,201,291,462,463,470,471,472,473]. These controversies are occurring in MEN1/ZES-MEN1 patients for a number of reasons. First, numerous pathology studies have demonstrated that 95–100% of MEN1 patients with or without ZES possess NF-pNET, which are almost invariably multiple, frequently microscopic, with only up to 13% estimated to cause symptomatic disease [290,328,371,474,475,476]. These occur relatively early in life in these patients, with penetrance estimated to start as early as age 10 [58] with 42% of the MEN1 patients already having pNETs by the second decade of life [477]. Second, long-term studies of MEN1 patients show these NF-pNET, because of their multiplicity and small size, are not completely resectable without a total pancreatectomy, which is rarely performed [475,478,479]; that pNETs, including NF-pNET, are the leading cause of death in these patients and contribute to their shortened life-expectancy [290,328,388,420,480]; that most of these small NF-pNET increase minimally or slowly with time [280,328,373]; that there are limited methods to assess which will become more aggressive [280,291], and most patients do well with NF-pNET ≤ 1.5–2 cm in diameter without surgical resection [366,374,481,482,483]. The result of this is the surgical treatment of asymptomatic, small (i.e., <2 cm); NF-pNET in MEN1 patients are controversial and re frequently postponed and replaced by a watch-and-wait approach. This will be dealt with in more detail in a later paper on surgical treatment of NF-pNET in MEN1 patients. Third, in addition to the NF-pNET in MEN1 patients, 20–71% of MEN1 patients have ZES/MEN1 [290], which is primarily due to the presence of duodenal gastrinomas (85–95%) which are invariable multiple, small (<0.5 cm), and frequently metastasize to adjacent lymph nodes (40–60%) [2,288,477,480,484]. Similarly to NF-pNET in these patients, the multiplicity of small duodenal gastrinomas associated with lymph node metastases results in the finding that the surgical cure rate by the recommended operations results in a low cure rate (<5%) [55,369,485] without aggressive surgical resections such as a Whipple resection, which are not recommended [46,365,366,372,486]. With the recommended, non-aggressive resections, most ZES/MEN1 patients with small tumors (<1.5–2 cm) and adequate acid secretory control have an excellent prognosis, which has led to controversy in their treatment, with many now managed non-surgically by a watch-and-wait approach [46,54,58,366,377,482,483,486,487].

- Controversies of the Roles of Imaging/Tumor Localization in MEN1 Patients for Gastrinomas/pNETs in Their Initial/Follow-Up Management
  – Controversy of the Role of Specific NET Imaging Studies in ZES/NET/pNET Diagnosis

As discussed in Section 2.2 above there is significant controversy in how, at present, to diagnose ZES, with the general abandoning of the classical criteria of assessing gastric pH/output combined with assessment of FSG with or without secretin provocative testing. This occurred primarily because of lack of availability of gastric fluid pH/analysis and the need to stop PPI therapy and its possible dangers in ZES patients (see Section 2.2 above). Some of the new possible diagnostic guidelines propose that one of the criteria that could be used for ZES diagnosis, after finding the presence of fasting hypergastrinemia (FSG) when the diagnosis is suspected, is to use various imaging study results, particularly, endoscopic ultrasound (EUS) evaluation of the duodenal pancreatic area [16,291,488], the presence of a positive somatostatin receptor imaging (SRI) study (^68^Ga-DOTATATE PET/CT) [226,489,490,491,492], or a positive conventional imaging studies (CT/MRI) [489]. In general, this approach is proposed but is controversial and uncommonly used and is not an established method of diagnosing ZES. This controversy exists because it has not been prospectively studied and the findings of these imaging studies are not specific for gastrinomas but can be seen in other NETs or even with non-NET pathology that can result in a positive imaging result. Furthermore, in patients with MEN1-ZES/MEN1 this approach can be a particular problem because these patients can develop multiple NETs in other tissues, which can be positive on SRI, as well as developing an increasing number of non-NETs which can be positive on imaging.

– Controversy of the Role of Specific NET Imaging Studies in NET/pNET Management in ZES-MEN1/MEN1 Patients

These controversies have developed primarily because of the uncertain role of surgery (both timing and type) in the management of both gastrinomas and small, NF-pNET, (<1.5–2 cm) in MEN1/ZES patients, or MEN1 patients in general, which was discussed above in Section Controversy of the Role of Specific NET Imaging Studies in ZES/NET/pNET Diagnosis. The controversies involve two areas—first, what imaging to perform initially in ZES/MEN1 or MEN1 patients and what imaging to perform regularly during follow-up. Important factors in determining this, but are in large part controversial, include the need for imaging for possible surgical resection, the evaluation of the extent of tumor involvement, the possible tumor progression with time either while following the patient in a wait-and-watch approach or while receiving antitumor treatments.

With ZES/MEN1 for assessment of the gastrinoma, almost all guidelines recommend an initial conventional cross-sectional imaging study (CT, MRI) when the ZES diagnosis is established [18,54,201,226,326]. Because the diagnosis is established and it is known that 80–95% of gastrinomas in ZES/MEN1 patients occur in the duodenum, which are small in size, associated with lymph node metastases in (40–60%) [2,288,477,480,484,493] and the conventional imaging studies frequently miss most pNETs < 1.5–2 cm and some >1.5–2 cm [54,226,291], guidelines also recommend that a ^68^Ga-DOTATATE PET/CT scan also be performed [18,54,226,326]. This allows localization of small duodenal lesions/accompanying lymph node metastases and assessment for more distant metastases, as well as assessment for other MEN1 accompanying NETs (carcinoids [lung, thymic, gastric], NF-pNET, meningiomas) [18,226,326]. The use of endoscopic ultrasound (EUS) in ZES/MEN1 is controversial because it detects <50% of duodenal lesions but is the most sensitive modality for detecting pancreatic NETs or pancreatic gastrinomas (the latter occurring in <5–20%) [18,226,494,495,496].

With ZES/MEN1 patients, in addition to assessment of the gastrinoma, similar to all MEN1 patients, assessment of the NF-pNET, that all MEN1 patients develop, needs to be performed [291,497] and complicates the management of the gastrinoma. Similarly to initial assessment of the gastrinoma, all guidelines recommend an initial conventional imaging study (CT, MRI) to assess the possible presence and size of NF-pNET in the ZES/MEN1 patient [54,291,326]. Generally, MRI imaging is recommended over the use of CT scan, because of the likelihood that repeated imaging will be needed during follow-up [58,326,498]. This occurs because of the inability to completely surgical remove all of the NF-pNET without unacceptable aggressive surgery, and the inability to predict their subsequent growth behavior, hence all these patients have a need for long term follow-up with repeated imaging, raising the question of possible deleterious effects of radiation in MEN1 patients, because of the known increased sensitivity of MEN1 containing cells to radiation [328,462,499,500,501,502]. Numerous studies show that ZES/MEN1 and MEN1 patients with imagined pancreaticoduodenal tumors < 1.5–2 cm have an excellent prognosis and this is commonly used as a criterion for whether routine surgical exploration is recommended [58,366,374,481,482,503]. However, in many cases of ZES/MEN1 this imaged lesion at surgery is not found to be the primary gastrinoma, which are more often, small; duodenal NETs are not seen on imaging, but are in fact metastatic gastrinomas in a lymph node, or a NF-pNET [5,225]. Both the frequency of follow-up revaluation is contentious as is the role of other imaging modalities in the long-term follow-up of both ZES/MEN1 and MEN1 patients is unclear in regard to the use of EUS, repeat ^68^Ga-DOTATATE PET/CT scan, or the use of ^18^F-Deoxyglucose scanning (^18^F-DG) [201,498,504,505,506,507,508]. The use of ^18^F-DG is recommended by some, because it is preferentially taken up by more aggressive grades of gastrinomas/pNETs and thus has been shown to have prognostic value in gastrinomas/pNETs/NET [291,509,510,511,512,513,514,515] by identifying more aggressive NETs that should be removed, rather than a further watch-and-wait approach. The controversy of what type of imaging to use in follow-up of patients with MEN1/ZES or MEN1 will best be resolved by prospective studies of these patients with a better definition of the natural history of these patients and identification of predictive factors which will allow definition of when interventions should be performed and what type.

### 2.4. Controversies Related to Non-Surgical Tumor Ablation for Treatment of ZES/Gastrinomas

#### 2.4.1. Background for Controversies Related to Ablation for Treatment of ZES/Gastrinomas

Numerous studies recently report the successful treatment of both F-pNET and NF-pNET by NET ablation performed endoscopically or percutaneously, primarily in patients with sporadic pNETs, but also including a few patients with MEN1 [516,517,518,519,520,521,522,523,524]. Particularly, recently receiving considerable attention has been the use of endoscopic ultrasound guided [525,526] techniques such as using ethanol injection or radiofrequency ablation (RFA) [525,527]. For F-NETs this includes primarily insulinomas; however, it also appears effective in other F-pNET, although few have been studied (i.e., <5 gastrinomas/VIPomas/ACTHomas) as well as NF-pNET [488,516,522,524,527,528,529,530,531,532,533,534,535,536,537,538,539,540,541]. Using these ablative techniques, pNETs were successfully treated in all regions of the pancreas including pancreatic head, body, and tail, as well as the uncinate process, with an overall clinical response rate of 88.9% reported in one systematic analysis involving ten studies (115 patients) [516]. The overall adverse events rate in this review [516] was 6.9% with the most common complication being acute pancreatitis (3.4%) followed by pancreatic duct stenosis, peripancreatic fluid collection, and ascites (2.8%) each [516].

In recent meta-analyses [527,542] of eleven EUS-RFA studies involving 292 patients with a minimum follow-up of at least 1 year, a complete radiological response was seen in 87.1% and a partial response rate in 11.4%, and for the F-pNET group only, the clinical response rate was 95%. The total adverse response rate was 20% with no severe event rate and no mortality. A metanalysis [525] compared results from studies using endoscopic RFA (EUS-RFA) and ethanol injection (EUS-EA) which included 100 EUS-RFA-treated pNET patients and 81 EUS-EA patients from 20 studies, showing no difference between the two ablation techniques in the technical success rate (94% vs. 97%), clinical success rate (85% vs. 82%), or adverse response rate (11.4% vs. 11.5%). In this meta-analysis [525] the location of the pNET in the pancreatic head/neck was a positive predictor for clinical success after EUS-RF [525], whereas in another systematic analysis [520], an increasing size of the NET was predictive for lack of ablative success with EUS-RFA, with ROC analysis finding a pNET size cut-off value of <18 mm predictive of response to treatment with a sensitivity of 80% and specificity of 79%. In this systematic analysis [520], there was no difference in the ablative success value of EUS-RFA in treating patients with F-NETs or NF-NETS.

#### 2.4.2. Controversies Related to Ablation for Treatment of ZES/Gastrinomas

The high success rate combined with a low complication rate of the above nonsurgical methods for ablating both NF-pNET and insulinomas has led to recommendations that this approach should be more widely applied [16,31,516,517,518,519,520,522,524,527,528,530,531,532,533,537,538,540,541,542,543,544]. In these papers, as well as in some guidelines/expert reviews, there is general agreement that this approach could be particularly valuable in patients with insulinomas/NF-pNET, in whom direct treatment of the pNETs is deemed necessary, and it is occurring in a patient who is a nonsurgical candidate or refuses surgical intervention. However, their routine utility in the treatment of patients with other pNETs or other clinical situations is unclear/controversial at present, for a number of reasons. First, in other F-NET syndromes (ZES, VIPomas, glucagonomas, etc.) its potential use has not been well studied, with only a few cases of ZES or VIPoma, one ACTHomas, and no others. The experience with insulinomas may not be applicable to these other F-pNET, because insulinomas are usually solitary, intrapancreatic, and without metastases, whereas the other F-NETs are frequently malignant with lymph node/hepatic metastases; in the case of gastrinomas they are primarily duodenal and very frequently small in location with lymph node metastases, all of which may affect ablative efficacy. Furthermore, there is excellent treatment for gastric acid hypersecretion in ZES, so that it may not have the clinical urgency of control of the hormone excess state that occurs with insulinomas or VIPomas. Second, in the above studies, most of the patients had sporadic pNET disease, with only a few patients with MEN1 with insulinomas/NF-pNET, so the applicability of these ablative techniques in MEN1 patients with other F-NET, or even with NF-NETs/MEN1, is unclear and the success rate could be markedly effected by the multiplicity of the these pNETs in these patients, as well as the fact that in those with ZES, the most frequent F-NET syndrome in MEN1 patients [290], the gastrinomas are almost invariably multiple in the duodenum, small in size (<0.5 cm), and are associated with lymph node metastases [334,335,337,338]. At present, for the reasons outlined above, it is not clear what role these nonsurgical ablative techniques in many pNET patients have, including those with ZES. Only future prospective studies will help establish the possible role(s) these nonsurgical ablative treatments may have in the management in ZES patients and even in other F-NETs patients, and in the management of both F-pNET and NF-pNET in MEN1 patients.

### 2.5. Controversies Related to Medical Treatment Selection for Advanced, Metastatic Disease in Patients with ZES/Gastrinomas/Other Malignant pNETs

#### 2.5.1. Background Related to Controversies Related to Medical Treatment Selection for Advanced, Metastatic Disease in Patients with ZES/Gastrinomas/Other Malignant pNETs

Gastrinomas are similar to other pNETs in that 50–80% are malignant and thus differ from insulinomas, which are malignant in <5–10% in most series [5,543,545,546,547,548]. Increasingly, in patients with non-insulinoma F-pNET syndromes, such as those with gastrinomas, the natural history of the NET and its malignant behavior are becoming the primary determinant of long-term survival [25,29,35,328,549,550,551]. This conclusion is well shown by the recent survival data for pNETs which reported median survivals for pNET patients with localized, regional, or distant disease for 1310 patients in the SEER database was 124, 70, and 23 months [552]. This occurs because of the increased ability to control the hormone excess state which was the leading cause of death in the past in many patients with F-PNET, which is now possible in 95–100% of all ZES patients because of the availability of potent acid antisecretory drugs such as PPIs [33,59,225], and in other malignant F-NETs because of other therapies such as PRRT, everolimus, telotristat, etc. [13,15,543,553,554]. Therefore, there is an increasing need for effective anti-tumor treatment in patients with advanced gastrinomas and other malignant pNETs. In patients with advanced well-differentiated pNET disease (i.e., grades G1, G2, G3 NETs) a number of different anti-tumor therapies have been used (Table 3).

This is in contrast to patients with poorly differentiated G3 neuroendocrine carcinomas (2–3% of all pNETs) [555,556], who are generally treated with chemotherapeutic regimes which involve platinum-based chemotherapy combined with either etoposide or in combination with other agents [557,558,559,560]. Although a number of controversial issues in both the surgical [11,369,561,562,563] and the nonsurgical treatment of patients with well-differenced advanced pNETs have occurred, one of the most important issues is the question of the order of use of the different anti-tumor medical approaches that are listed in Table 3 [547,548,564,565,566,567,568,569].

#### 2.5.2. Controversies in the Nonsurgical Treatment Selection Order in Patients with Advanced, Metastatic Disease with ZES/Gastrinomas or Other Malignant pNETs

There are no studies with only patients with advanced gastrinomas that directly address this question because of their rarity. Because this issue with patients with malignant advanced gastrinomas is the same as with patients with other malignant pNETs, and also to a large degree, with any advanced GI-NET, the studies that provide some insight into this issue are from ones that include gastrinomas with other malignant pNETs or other GI-NETs [547,548,564,565,566,567]. Almost all guidelines and expert reviews of this subject recommend the use of somatostatin analogs as the initial antitumor treatment for most patients with well-differentiated gastrinoma/pNET patients, if they show positive uptake on ^68^Ga-DOTATATE PET/CT [57,392,467,543,570]. These drugs are well tolerated, have been shown to prolong time to progression/progression free survival/in patients with pNETs/NETs in prospective studies [571,572,573,574]. However, they are almost entirely tumoriostatic in action with tumor stabilization occurring in various studies in 40–80% [575,576], with the rate of decrease in tumor size with their use being low (<5–10%). The tumor stabilization can be prolonged in some cases for >2 yrs [394,577]. Some studies show that the tumoristatic effect of somatostatin analog is more frequent in slower growing tumors with lower proliferative rates and thus recommend that patients with more rapidly growing tumors or those with higher proliferative rates, or evidence of aggressive growth with bone metastases [578], should be initially treated with other modalities [394,548,573,576,579]. If treatment with somatostatin analogs fails, the best approach at present is unclear because of lack of prospective studies and is thus controversial for most patients [547,548,565,566].

Numerous guidelines, especially for advanced well-differentiated pancreatic neuroendocrine tumors recommend targeted therapy and chemotherapy as the preferred treatment of choice, and the use of PRRT has been recommended only after failure of these therapies [556,580,581,582,583]. Furthermore, guidelines from ENETs [57], European NET Society [556,582,583], NANETs [581], and the National Comprehensive Cancer Network [582] recommend chemotherapy as the primary treatment. However, physician groups may vary in their treatment sequences (SSAs and/or chemotherapy and/or everolimus or sunitinib as first- and second line therapies) and the use of these agents as first-/second line therapy can be individualized. The exact role of PRRT and correct timing is still generally unclear.

Recently, because of the efficacy of PRRT in controlling the hormone excess state of a number of F-NETs/F-pNET for which there are not optimum therapies, which is not the case with gastrinoma, but includes including VIPomas, malignant insulinomas, PTHrPomas and ACTHomas [543,553,554,575,584,585,586,587,588,589,590], it is recommended in these patients if the hormone excess state control as well as tumor growth are both problems, then PRRT should be recommended over chemotherapy or molecular targeted therapies [548]. For the majority of patients who fail somatostatin analogs (SSA) for treatment of their advanced disease there is no well-established sequence that all agree on. One recent consensus conference [548] recommended in asymptomatic patients with advanced disease from a G1 pNETs with low tumor burden and progression on SSAs that the second line treatment should be either everolimus or sunitinib (88% consensus) primarily based on the results of the RADIANT-3 Phase 3 randomized control trial (RCT) [591] and the sunitinib Phase III RCT [592], which both showed highly significant increases in PFS with everolimus or sunitinib treatment of patients with advanced pNETs. In this same consensus conference [548] in patients with advanced G1 functional pNETs, with SSTR expression, and progression on SSAs, the use of PRRT was recommended, which is now supported by results from a number of studies [593,594,595]. In this same consensus conference [548] 62% of the participants recommended using at least one SSA, targeted therapy, or chemotherapy (streptozotocin or temozolomide-based) before PRRT in advanced G1-G2-pNETs with SSTR expression and 96% recommended chemotherapy prior to PRRT in G2 NF-pNET with high tumor burden and symptoms which was supported by a number of other reports [596,597,598,599,600]. In high burden cases with pNETs, some experts recommend chemotherapy as the preferred option [556,600,601].

In contrast to the above guidelines, some [467] recommend that PRRT should be first line treatment for any patient with grade 2 GEP-NET with Ki67 > 10% or grade 3 GEP-NET based on results of NETTER-2 study [602]. At present the current generally used standard of care for patients with GEP-NETs with Ki67 of 10–55% is SSAs unless high tumor burden or symptoms related to growth are present [603]. Others recommend PRRT as first line systemic treatment in all patients with advanced inoperable/metastatic NETs [604]. In a recent retrospective, multicenter study [605], results were compared in 508 patients who demonstrated tumor progression on treatment with somatostatin analogs, with advanced NETs (260-pNETs, 248-intestinal), in which 65% received upfront PRRT or 35% upfront chemotherapy or targeted therapy. Median PRS was longer in the PRRT group vs. chemotherapy or targeted therapy (2.5 vs. 0.7 yrs); however, there was no difference in overall survival [605]. The increased PFS was independent of site of origin of NET, functionality of NET, or NET grade (1 vs. 2).

The overall therapeutic efficacy of ^177^Lu-DOTATATE in advanced NETS from an analysis of 22 studies (1758 patients) was a disease response rate complete response (CR), partial response (PR), or stable disease (SD) of 79% with a disease control rate (CR, PR) of 33% by RECIST criteria [606]. In 610 patients [607] with Gr 1 or 2 advanced GEPNET/bronchial tumors treatment with ^177^Lu-DOTATATE with a mean follow-up of 64 mos., the median PFS after PRRT was 30 mos. for pNETs and midgut NETs, and the median overall survival was 71 and 60 mos., respectively, with associated toxicity similar to the NETTER-1 study [608,609] and other studies of 4.7–9% hematological adverse Gr3/4 events [610]. PRRT results with ^177^Lu-DOTATATE in less than 50 patients with metastatic gastrinomas have been reported [585,594,611,612,613,614,615,616,617] with a PR rate of 45%, but the time to progression was shorter than that in NF-pNET [594]. Whether these responses will be better than those to chemotherapy or targeted therapy in these patients, without the added benefit of the effect of PRRT on the hormone-excess state, which is well-controlled in these patients with PPIs, is at present unclear. At present, the best sequence of anti-tumor treatment for patients with advanced pNETs including patients with metastatic gastrinomas in a number of the scenarios reviewed above remains controversial and unclear and will only be resolved by direct comparative studies.

## 3. Discussion

In this paper the basis and current status of seven medical aspects which are controversial in the management of patients with ZES are reviewed and discussed. Specifically, these include controversies related to the safety and feasibility of long-term control in ZES patients of the acid hypersecretion with the increasing reported acid antisecretory drug side-effects/antisecretory drug failure rates; controversies related to the difficulty in making the diagnosis of ZES; three controversies related to the nonsurgical aspects of management of the 25% of patients with ZES/MEN1 including the management of gastric carcinoids (Type 2) in MEN1/ZES patients, controversies of whether genotype-phenotype correlations exist in MEN1 patients including MEN1/ZES patients and controversies of the role of specific NET imaging studies in NET/pNET diagnosis/localization in ZES-MEN1/MEN1 patients; controversies related to the possible role of non-surgical tumor ablation for treatment of ZES/gastrinoma; and controversies related to the possible sequence of medical treatment for advanced, metastatic disease in patients with ZES/gastrinomas/other malignant pNETs. Most of these controversies are unique to the management of patients with ZES including those related to control of the acid hypersecretion; controversies related to the diagnosis of ZES; each of the three MEN1/ZES controversies, and controversies related to the non-surgical primary NET ablation for treatment, whereas controversies related to the selection sequence of anti-tumor medical treatments in patients with advanced disease is shared by patients with all malignant pNETs and in many aspects with the management of patients with any advanced NET. The controversy involving the role in ZES patients of the non-surgical ablation of the primary NET (gastrinoma) for treatment is unique because in contrast to a number of other F-NETs, the hormone excess state effect resulting in gastric acid hypersecretion due to the ectopic gastrin release, can be very effective control in most patients with the potent acid antisecretory drugs available, such as the PPIs [6,33,59,60,61,62,63,64,65,66,67,68,69,70,382]. In contrast, in patients with localized disease that are not surgical candidates with VIPomas, insulinomas, carcinoid syndrome, ACTHomas, and rarely glucagonomas, the medical treatment of the hormone-excess state is frequently inadequate, and the use of nonsurgical ablation of the primary tumor is an increasingly used option [488,516,522,524,527,528,529,530,531,532,533,534,535,536,537,538,539,540,541,553]. Therefore, in ZES the need for ablating the primary tumor to control the hormone-excess state is not a major consideration, whereas it can be for patients with the other F-NET syndromes listed above. This is in contrast to the use of ablative techniques (i.e., RFA, microwave ablation, cryoablation, laser-ablation, etc.), used at the time of surgery or percutaneously in patients with metastatic disease, primarily in the liver, to control the tumor growth, which is widely used in patients with metastatic gastrinomas [618,619,620,621,622,623,624] as well as other malignant pNETs/NETs [620,621,622,623,624,625,626,627].

## 4. Conclusions

A summary of the principal points regarding the current staus of these medical controverises and their possible resolution discussed in the previous paragraphs is shown in Table 4.

The resolution of each of the seven medical controversies in the management/treatment of ZES patients reviewed and discussed in detail in this paper will likely markedly vary in time. Meanwhile, the controversy related to the selection sequence of anti-tumor medical treatments in ZES patients with advanced disease, which is shared by patients with all malignant pNETs/NETs, will likely be resolved relatively quickly, the remaining six controversies, which are specific for ZES, will likely take longer to be fully resolved, if resolved at all. The difference in the time of resolution is in large part due to the availability of patients to study the different controversies. As discussed above in Section 2.5, numerous studies are already exploring the sequence order of anti-tumor medical treatments in patients with advanced pNETs/NETs. This includes different groups of patients with different orders of treatments, which allow identification of subsets of patients who will benefit more from one sequence of medical treatments than another. Even though patients with GI NETs (Carcinoids) and pNETs can differ markedly in their response to various chemotherapy/tyrosine kinase inhibitors, etc.) [628,629,630,631,632], which could affect the sequence order of treatment with these two types of NETs, sufficient numbers of patients are available, so that, as reviewed in Section 2.5, studies are already providing important insights into the comparative effects of different sequencing schedules, particularly with regard to PRRT [467,564,568,593,602,605,632,633,634,635]. In contrast, in six ZES-specific controversies, it is likely that the time line to resolve these will be longer, even though, as reviewed above, both the recognition of these areas as needing attention, as well as number of current studies providing possible insights into the resolution of these controversies are already available, to actually establish these solutions as standard practice will require for each ZES controversy, systematic studies of ZES patients with different treatment/diagnosis/approaches that will need to be performed, and few, if any, centers, have sufficient ZES or ZES/MEN1 patients to do these studies alone. Therefore, these studies will need to be performed as multicenter studies and even this approach will be hindered by patient numbers and availability of resources. For example, the lack of gastric analysis/pH testing in most centers will make any study done on assessing the effectiveness of different antisecretory treatments difficult, as well as any study on diagnosis that involves altering the PPI antisecretory dose. Furthermore, in the case of ZES/MEN1 patients, the controversial role of surgery or the type of surgery in these patients for the gastrinomas or NF-pNET will complicate the design of any study to address the other controversial issues in these patients discussed here.

## Figures and Tables

**Table 1:** Classical and currently used methods for the diagnosis of ZES.

**Classical approach for ZES diagnosis** [6,18,54,206,225,226] **(at present used <5% of all reported cases) ^(1)^** A.1. If ZES is suspected clinically, a **fasting serum gastrin (FSG)** determination in the absence of antisecretory therapy is the recommended initial diagnostic study [227]. A.2. An elevated FSG is not diagnostic of ZES because it can be caused by physiological (response to hypo-/achlorhydria) and numerous pathophysiological conditions. An increased FSG can only be accurately interpreted for the possible diagnosis of ZES if it is obtained at a time when some measure of gastric secretory capacity (usually **a gastric pH**) is performed. The detection of an elevated FSG in the presence of an acid pH < 2 establishes the presence of inappropriate hypergastrinemia which is needed for ZES diagnosis. In almost all patients an accurate assessment of gastric pH can only be performed after either delaying or stopping gastric antisecretory treatment. A.3. If the **FSG is >10-fold increased** at a time when **the gastric fluid pH is ≤2,** the diagnosis of ZES can be established. The rare postsurgical condition of retained gastric antrum syndrome [222] can also fulfill this criteria; however, it can be almost always ruled out by a careful history. (This combination of FSG > 10-fold increased and gastric pH ≤ 2 was found in 32% of a large series of ZES patients [227,228].) A.4. If the **FSG is increased <10-fold** and the gastric pH is ≤2, which occurs in 68% of ZES patients [227,228], additional test are generally recommended; they include either a secretin provocative test or a full determination of basal acid output (BAO), which is now rarely available. A.5. If the **secretin provocative test** results in an increase in FSG of >120 pg/mL a diagnosis of ZES is established with a sensitivity of 94% and specificity of 100% for ZES [229]. In the rare case where full gastric acid testing is available, the finding of a **BAO > 15 mEq/h** in a patient with no previous gastric surgery or >5 mEq/h with a previous history of gastric acid reducing surgery, combined with the FSG/pH changes listed in IV above, establishes the diagnosis of ZES [47,48,227]. **Currently used approach ^(2)^** B.1. Initially, ZES was first suspected by clinical history usually involving refractory PUD/GERD, often with a PUD/GERD disease complication (perforation, bleeding, stricture, etc.), **leading to determination of FSG** [230,231,232]. In some cases, there is further confirmation that it may be a positive conventional imaging study (CT, MRI) showing an abdominal/pancreatic mass or a positive somatostatin receptor imaging (SRI) study (^68^Ga-DOTATATE PET/CT or ^111^In-DTPA-ocreotide with SPECT/CT imaging) or less frequently a percutaneous biopsy (liver, etc.) showing gastrinoma.

^(1)^ Recommended approach text modified from [203]; ^(2)^ Currently used approach from review of 20 ZES cases reported in the literature in [203] over the 4 years from 2013 to 2017 [106,112,113,233,234,235,236,237,238,239,240,241,242,243,244,245,246,247] as well as 15 case reports reviewed from 2018 to 2025 [44,96,97,99,100,105,108,115,117,118,248,249].

**Table 2:** Studies/reviews investigating genotype–phenotype correlations in MEN1 patients.

Author, yr (Ref)	# pts	Exon	Type Mutation	Association
**ZES Only/** **ZES Related**				
Ito, 2013 [328]			Frameshift-insertion/deletion	Higher rate in MEN1/ZES patients alive than dead (*p* = 0.039)
Men1 Burin variant (Olefumi, 2004 [406], Hao, 2004, Kong, 2001 [407])			Nonsense mutation (TYR312stop, ARG460stop)	Have high frequency of prolactinomas and carcinoids, low rate of gastrinomas
Vierimaa, 2007 [408]	82	10 1466del12, 1657 insC	In-frame deletion of exon 10 amino acids 453–456	Ex10 1466del12 associated with higher rate of gastrinoma
**All MEN1 pts**				
Agarwal, 1997 [403]	58			No genotype–henotype correlation
Giraud, 1998 [409]	84			No genotype–phenotype correlation
Bassett, 1998 [402]	195			No genotype–phenotype correlation
Poncin, 1999 [410]	25			No genotype–phenotype correlation
Bartsch, 2000 [53]	19	2, 9, 10	Truncating frameshift or nonsense in C-/N terminal	Increased risk of malignant pNET
Kouvaraki, 2002 [411]	109		Frameshift	Frameshift mutations more frequent in pts with PETs (*p* = 0.03). Type/site of mutation did not correlate with metastatic disease
Wautot, 2002 [412]	170 families			No genotype–phenotype correlation
Machens, 2007 [413]	258			No genotype–phenotype correlation
Vierimaa, 2007 [408]	82	10 1466del12, 1657 binsC	NF-PNET = Frameshift/nonsense mutation inc 3.3x; 1657insC = Inc 3.6. Gastrinoma = In Frame/Missense Inc6.8X	-NFpNET inc by frameshift/nonsense mutations or 1657insC inc 3.3 -Gastrinomas inc 6.6 x by in-frame/missense mutations
Lemos, 2008 [400]	Men1 pts with 1336 mutations			No genotype–phenotype correlation
Thakker, 2010, 2013 [321,414]	Review MEN1			No genotype–phenotype correlation
Sakurai, 2012 [415]	419			No genotype–phenotype correlation
Thevenon, 2013 [416]	806		Jun D interacting site	No genotype–phenotype correlation. Pts with mutation affecting JunD interacting site had higher risk of death
Bartsch, 2014 [417]	71	9, 10	Mutation resulting in loss of interaction with CHES1 EX 9,10	Associated with higher rates of malignant F-pNET, pNETs with distant mets, and pNET death
Christakis, 2018 [405]	188	2		Higher risk malignant pNET and pNET with distant mets; Higher risk pNET for pts 20–40
Marini, 2018 [404]	410		nonsense	GEP-NETs more freq in pts with nonsense mutation. Th-NETs had higher % with spicing-site mutation
Kovesdi, 2019 [418]	47		Frameshift, nonsense, splice-site, large deletion	More freq developed GEP-NETs
Thevenon, 2018 [404]	797			No genotype–phenotype correlation
Soczomski, 2021 [419]	63	2		Increased risk of pNET with metastases
			Frameshift, splice site, missense	Less advanced disease
		5		pNETs diagnosed earlier
Gaugoux, 2022 [420]	1386	2	Mutation affecting JunD interaction	Associated with decreased survival (*p* < 0.001)
Ramamoorthy, 2023 [421]	Review, studies of genot–phenot correlations			No genotype–phenotype correlation firmly established
Worthy, 2025 [422]	162 [147-Genotype+ MEN1 pts, 47-Gentoype neg MEN1 pts]			Genotype + MEN1 pts had higher rate duopan NETs; ZES than genotype neg MEN1 pts
		2		Genotype + MEN1 pts with mutation in Ex2 had lower rate of distant mets
Kim, 2025 [423]	72		Truncating mutations	Age-penetrance higher with mutation (*p* = 0.029)
		3, 10	Any mutation	Inc tumor progression (*p* = 0.007)

Abbreviations: LN, lymph node; Panc, pancreatic; ex, exon; freq, frequency; pNET, pancreatic neuroendocrine tumor; genot–phenot, genotype–phenotype correlation; duopan, duodenopancreatic; mets, metastases; neg, negative; pts, patients; INC, increased.

**Table 3:** Treatment modalities used for advanced metastatic disease in patients with ZES/gastrinoma and other malignant pNETs/NETs.

Biotherapy Somatostatin Analogs Interferon Cytoreductive surgery Chemotherapy Molecular targeted therapies mTor Inhibitors (Everolimus) Receptor tyrosine kinase inhibitors (RTKs) [Sunitinib, surufatinib (China only)] Liver-directed therapies Radio-frequency ablation (RFA)/other locally ablative therapies Embolization/Chemoembolization Radio-embolization/selective internal radiation therapy (SIRT) Peptide Receptor Radionuclide Therapy (PRRT) Immune therapy Other: angiogenesis inhibitor [bevacizumab (VEGF inhibitor)]

**Table 4:** Summary of status (current/future needs) of unclear medical controversies in ZES discussed above.

Controversy	Current Status	Possible Resolution in Future
Control acid hypersecretion/anti-secretory drug side effects	Increasing reports of difficulty controlling active hypersecretion long-term. Increasing reports of acid antisecretory drug side-effects, VB12 def, hypomagnesemia, etc.	Increased referral of possible and established ZES patients to established NET/pNET centers with expertise in managing acid hypersecretion would greatly help. With prolonged treatment patients should be periodically assessed for vitamin/electrolyte/iron status and encouraged to take a daily MVI as well. Acid output testing in some specialty centers should be more widely available.
II. Difficulty in ZES Diagnosis (Dx)	Increasing reports of difficulty in ZES DX. Due to lack of acid secretory testing, long duration of action of PPIs.	Prospective studies of some newly proposed methods/criteria for making the diagnosis with limited or no acid testing need to be explored.
III. MEN1/ZES: Manage Type 2 gastric carcinoids	Usually watch/wait approach and/or removal of larger gastric polypoid lesions performed. Multiple lesions increase with time, and higher malignancy rate than type 1, increasing problem as no longer due to total gastrectomy. Natural history largely unknown.	Prospective studies of patients with Type 2 gastric carcinoids for natural history and treatment are needed.
IV. MEN1/ZES: Unclear genotype–phenotype correlations	No MEN1 genotype–phenotype correlation widely accepted/used. Unclear those described, if useful clinically.	Prospective studies of patients with various genotype–phenotype correlations showing positivity for various needed.
V. MEN1/ZES: Unclear pNET imaging sequence-initial, follow-up	Unclear what imaging studies should be routinely used initially or during follow-up of NF-pNET at different ages. No general agreement on managing small (<1–2 cm) NF-pNET and in MEN1/ZES.	Additional prospective studies of patients with small NF-pNET (<1–2 cm) need to be performed to establish natural history and best method to identify aggressive tumors. Role of EUS/FNA needs to be defined and identify which patients, if any, need it. Additional prospective studies of MEN1/ZES with small gastrinomas/NF-pNET followed without surgery need to be performed.
VI. Role of nonsurgical tumor ablation	Increasing reports of use of successful nonsurgical ablation of F-/NF-pNET especially in patients not able to undergo surgery. Unclear whether it should be more widely used; if so, what group; long-term success	Prospective studies of nonsurgical ablation should be extended to additional patients with any relative contraindication to surgery with careful follow-up with NF-pNET/F-pNETs.

## Data Availability

No new data have been reported or analyzed in this study.

## Abbreviations

The following abbreviations are used in this manuscript:

ACTH	ACTH, adrenocorticotropic hormone
AP-1	AP-1 Transcription Factor Subunit
AP1	AP1, activating protein-1
BAO	Basal acid output
CAG	CAG, chronic atrophic gastritis
CCK_B_	Cholecystokinin B receptor
CNS	Central Nervous System
CR	Complete response
CT	Computed tomography
CVS	Cardiovascular disease
ECL-cells	Enterochromaffin-like cells
ENET	European Neuroendocrine Tumor Society
EUS-EA	Endoscopic Ultrasound-guided Ethanol Ablation
EUS-RFA	Endoscopic ultrasound guided radiofrequency ablation
EUS	Endoscopic ultrasound
F-pNET	Functional pancreatic neuroendocrine tumor
FANCD2	Fanconi anemia, complementation group D2
FSG	Fasting Serum Gastrin
GERD	Gastroesophageal reflux disease
GI-NETs	Gastrointestinal neuroendocrine tumors
GI	Gastrointestinal
H_2_R	H_2_-receptor antagonist
JunD	Jun D Proto-Oncogene
LH	Linear hyperplasia
MAO	Maximal acid output
MEN1	Multiple Endocrine Neoplasia type 1
MENIN	Protein encoded by the MEN1 gene
MRI	MRI, Magnetic resonance imaging
NANET	North American Neuroendocrine Tumor Society
NET	Neuroendocrine tumor
NF-pNET	Non-functional pancreatic neuroendocrine tumor
NIH	National Institutes of Health
NM23	Nucleoside diphosphate kinase A
pNET	Pancreatic neuroendocrine tumor
PPIs	Proton pump inhibitors
PR	Partial response
PRRT	Peptide Receptor Radionuclide Therapy
pts	Patients
PUD	Peptic ulcer disease
RCT	Randomized control trial
RFA	Radio-frequency ablation
RTK	Receptor tyrosine kinase
SD	Stable disease
SIRT	Radio-embolization/selective internal radiation therapy
SMAD3	Mothers Against Decapentaplegic Homolog 3
SPECT	Single Photon Emission Computed Tomography
SRI	Somatostatin receptor imaging
SSA	Somatostatin analogs
tHCY	Total homocysteine
UD	Peptic ulcer disease
UGI	Upper gastrointestinal Series
ULN	Upper limit of normal
US	United States
VB_12_	Vitamin B_12_
ZES	ZES, Zollinger-Ellison syndrome
^111^In-DTPA-octreotide	Indium-111 labeled diethylenetriaminepentaacetic acid-octreotide
^177^Lu-DOTATATE	Lutetium-177 (^177^Lu)-dotatate
^18^F-DG	^18^F-Deoxyglucose scanning
^68^Ga-DOTATATE	Gallium-68 DOTA-DPhe^1^, Tyr^3^-octreotate
